# Proteomics and metabolic burden analysis to understand the impact of recombinant protein production in *E. coli*

**DOI:** 10.1038/s41598-024-63148-y

**Published:** 2024-05-28

**Authors:** Girish H. Rajacharya, Ashima Sharma, Syed Shams Yazdani

**Affiliations:** 1 Microbial Engineering Group, International Centre for Genetic Engineering and Biotechnology (ICGEB), Aruna Asaf Ali Marg, New Delhi, 110067 India; 2 DBT-ICGEB Centre for Advanced Bio-Energy Research, International Centre for Genetic Engineering and Biotechnology, New Delhi, India; 3 School of Interdisciplinary Research (SIRe), Indian Institute of Technology, New Delhi, India; 4 Present Address: Department of Life Sciences, J.C. Bose University of Science and Technology, YMCA, Faridabad, Haryana India

**Keywords:** Recombinant protein production, Metabolic burden, Gene expression, Proteomics, Acyl-(acyl carrier protein) reductase (AAR), Post-induction growth phases, Expression systems, Proteomics

## Abstract

The impact of recombinant protein production (RPP) on host cells and the metabolic burden associated with it undermine the efficiency of the production system. This study utilized proteomics to investigate the dynamics of parent and recombinant cells induced at different time points for RPP. The results revealed significant changes in both transcriptional and translational machinery that may have impacted the metabolic burden, growth rate of the culture and the RPP. The timing of protein synthesis induction also played a critical role in the fate of the recombinant protein within the host cell, affecting protein and product yield. The study identified significant differences in the expression of proteins involved in fatty acid and lipid biosynthesis pathways between two *E. coli* host strains (M15 and DH5⍺), with the *E. coli* M15 strain demonstrating superior expression characteristics for the recombinant protein. Overall, these findings contribute to the knowledge base for rational strain engineering for optimized recombinant protein production.

## Introduction

The recombinant proteins have a wide range of uses in various industries, such as pharmaceutical, chemical and biotechnological, for producing diverse molecules via biocatalysis. Various hosts are being exploited for producing recombinant proteins heterologously^1^. Nevertheless, the selection of an appropriate host for RPP remains a challenging task due to changes in the paradigm and dynamics of cellular processes of the host system. In this context, one of the most used microbial species for producing recombinant proteins is *Escherichia coli* due to its well-understood genetics and physiology, ease of manipulation, low cost of culture, and rapid growth^2^. Its further advantages include attaining high cell density, scalability, and well-established engineering tools to alter and optimize various parameters to regulate the cellular processes of the host and, in turn, RPP.

There are several cellular and environmental factors that affect the RPP in the host system. The type of media used for bacterial growth and choice of protein expression system are among the major parameters that influence the RPP in a very significant manner^3^. The promoter used for facilitating the expression of RPP is another factor that makes a large difference. There are two bacteriophage-derived promoters that have extensively been used for this purpose in *E. coli*—T5 and T7 bacteriophage promoters. The T5 bacteriophage utilizes the host RNA polymerase to transcribe its genes^4^. In contrast, T7 bacteriophage encodes its own T7 RNA polymerase for transcribing its genes. Therefore, RPP, under the control of the T7 bacteriophage promoter, needs co-expression of T7 RNA polymerase in the host^5^. While this strategy has the advantage of having a dedicated RNA polymerase enabling exclusive expression of genes under T7 RNA polymerase promoters^6^, its expression is limited to the host that co-expresses the T7 RNA polymerase gene. Therefore the utility of *E. coli* as a host for T5 promoter-controlled gene expression is much wider.

Higher quantities of expressed recombinant protein in an *E. coli* host leads to a notable impact on host cell metabolism detectable through growth retardation, which is generally known as “metabolic burden”^7,8^. The factors contributing to the metabolic burden are multifaceted, depending on the host/vector combination and on the properties of the encoded gene with its transcription and translation products, as well as on the environmental conditions encountered during the production process^9–11^. The prime reasons for the protein production-related metabolic burden have been attributed to plasmid amplification and maintenance, transcription, translation, and protein folding-related processes^12^. However, the production of the same protein under identical conditions in the same vector combination but in different hosts can change the metabolic perturbations considerably, suggesting that the phenomenon is even more complex than anticipated^13^. Insights of the metabolic potential of exponentially growing and stationary phase cells are critical not only for understanding and improving production processes but also for gaining knowledge and a better comprehension of bacterial physiology^14^. Considering *E. coli* as the host of choice for the production of numerous recombinant proteins, biopharmaceuticals, small molecules and homologous metabolites^15^, it becomes imperative to gain insights into the effect of RPP on the host system metabolism.

Often, the recombinant proteins expressed inside the heterologous host have certain catalytic functions, and the substrate (especially if it is synthesized inside the host) or the product of the catalytically active proteins may interfere with the host physiology. This is true for all metabolic engineering-related strategies. Our laboratory is involved in microbial hydrocarbon production in *E. coli* where cyanobacterial Acyl-ACP (acyl carrier protein) reductase (AAR) plays an important role^16^. We thus selected AAR as a reference recombinant protein in the present study. AAR expression impacts the host cell in a significant manner, and it is extremely difficult to express in active form because of the marginal stability exhibited by the protein when expressed in *E. coli* ^17^. Therefore, studying the impact of producing such tough protein on cellular metabolism will help to gain cellular insights because of the complexity involved with its expression and folding in the heterologous host.

In the current study, we used a pQE30-based expression platform that uses a T5 promoter for expressing recombinant protein, and through employing a label-free quantification (LFQ) proteomics approach, we have analysed the whole cell proteome of the cells expressing recombinant protein when subjected to different conditions and compared it with the control. Our results on proteomics investigation of the two commonly used *E. coli* hosts, i.e., M15 and DH5⍺, under different culture media (LB & M9) conditions, show significant differences in gene expression and cellular dynamics during the growth and protein production phase. Significant changes in DNA metabolism, RNA metabolism, transcription, translation, protein folding/secretion, sigma factors, cell division, and transporters were observed, which have significance in designing the host for high-level recombinant protein production.

## Results

### Identification of process parameters for optimal recombinant protein production

**Figure 1:**
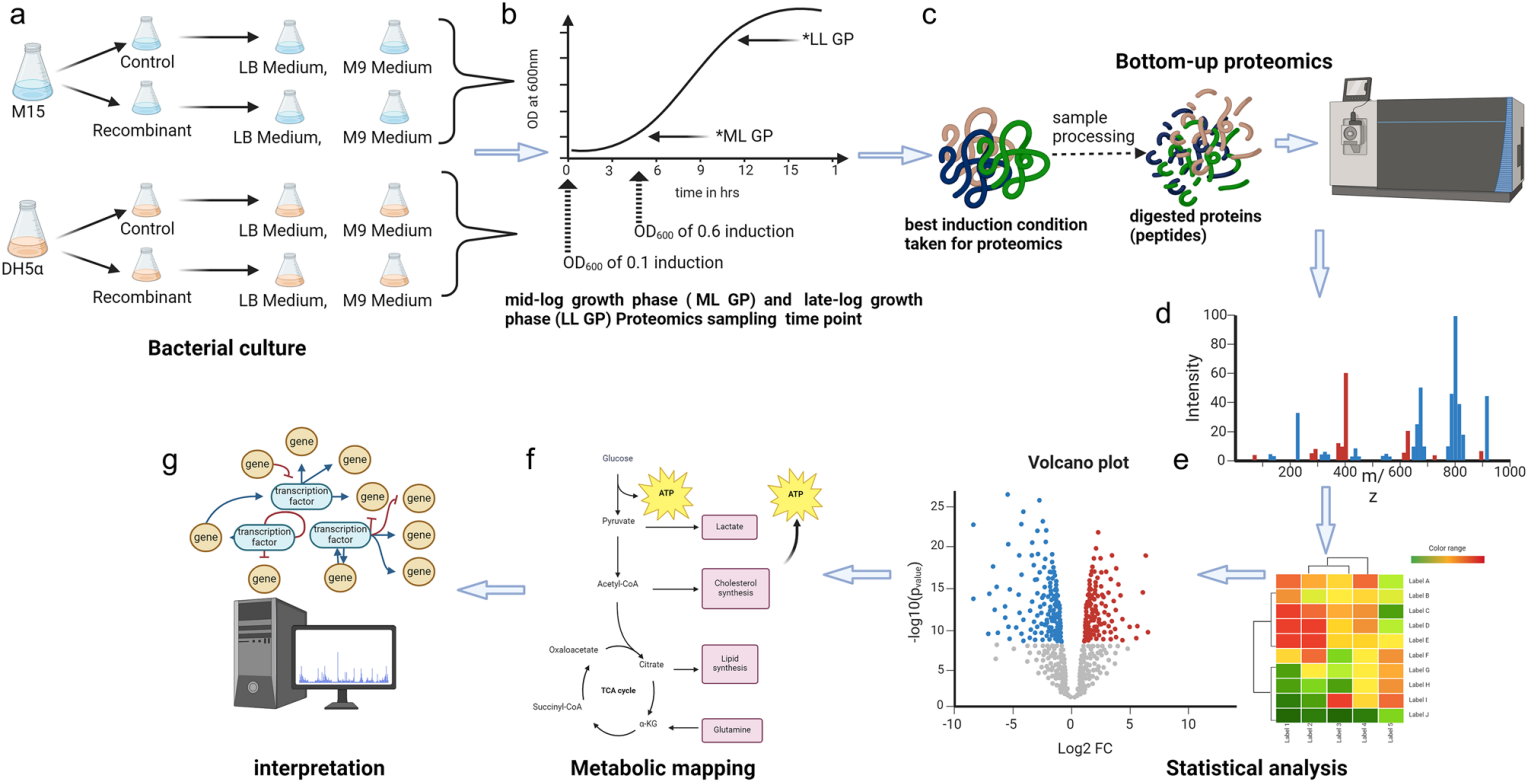
Schematic representation of study design. (**a**) culturing cells with two different hosts (*E. coli* M15 and *E. coli* DH5⍺), (**b**) growth profile with induction and proteomics sampling time point, (**c**) bottom-up proteomics experiment, (**d**) data analysis, (**e**) data shortlisted based on statistical approach, (**f**) metabolic mapping, and g. interpretation.

Several crucial cellular and environmental process parameters exist that play a key role in determining the fate of recombinant protein production in a host system^18,19^. A systematic study was designed to understand these phenomena as represented schematically in Fig. 1. The study includes investigating the effect of basic yet crucial parameters like host cellular environment (different host strains), protein-induction time point, post protein-induction duration, cell growth, growth medium, and end-product (the catalytic product of recombinant enzyme) on the expression of recombinant protein acyl-ACP reductase (AAR) in *E. coli* host. The induction time point for the proteomics study was chosen based on the optimal expression of the key protein (AAR).

**Figure 2:**
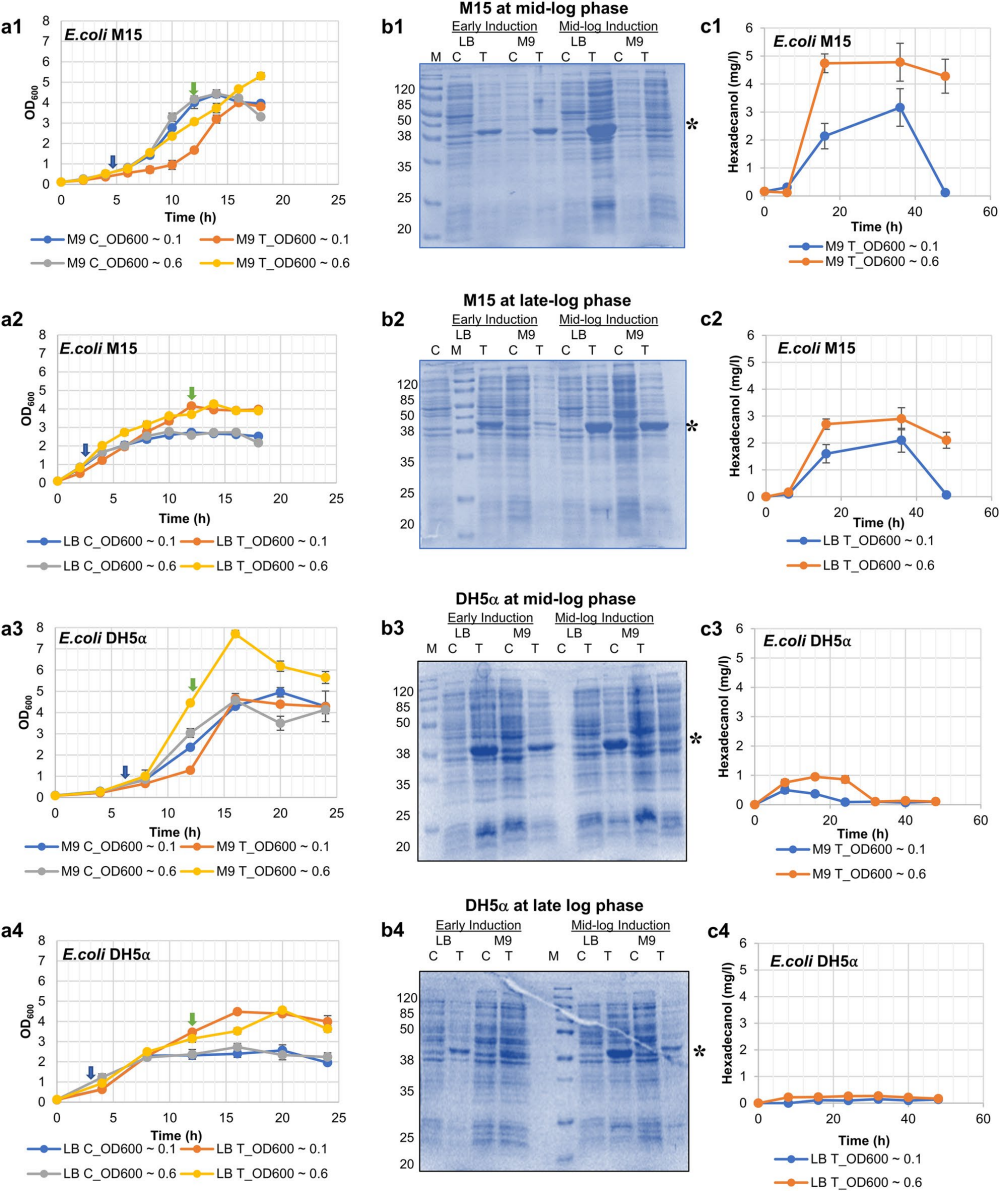
Comparative growth and protein and product profile of *E. coli* hosts grown in different media. (**a1**) *E. coli* M15 with M9 medium growth curve. (**a2**) *E. coli* M15 with LB medium growth curve. (**a3**) *E. coli* DH5⍺ with M9 medium growth curve. (**a4**) *E. coli* DH5⍺ with LB medium growth curve. (**b1**) SDS PAGE for *E. coli* M15 at post induction mid-log growth phase, (**b2**) SDS PAGE for *E. coli* M15 at late-log phase, (**b3**) SDS PAGE for *E. coli* DH5⍺ at post induction mid-log growth phase, (**b4**) SDS PAGE for *E. coli* DH5⍺ at late-log phase (Note-Full length gel available as Fig. S7). (**c1**) Hexadecanol Profile for *E. coli* M15 with M9 Test at early and mid-log induction. (**c2**) Hexadecanol Profile for *E. coli* M15 LB Test at early and mid-log induction. (**c3**) Hexadecanol Profile for *E. coli* DH5⍺ M9 Test at early and mid-log induction. (**c4**) Hexadecanol Profile for *E. coli* DH5⍺ LB Test at early and mid-log induction; LB = Luria Bertani medium; M9 = Minimal modified medium; C = Control; T = Test (Recombinant strain). (*) mark indicates recombinant protein Acyl-(acyl carrier protein (ACP)) reductase (AAR). Growth curve has been constructed using cell growth parameters measured via optical density at 600 nm (OD_600_). Blue and green arrow marks indicate the sampling time points for the mid-log phase and late-log phase, respectively.

The growth and end-product (hexadecanol) formation profile of two different *E. coli* host strains (M15 and DH5⍺) expressing recombinant AAR were compared with that of the control. The strains were cultured in two different growth media (defined M9 and complex LB), and recombinant protein was induced at two different growth points (early-log phase at OD_600_ of 0.1 (at the time of inoculation) and mid-log phase at OD_600_ of 0.6). This resulted in 8 different sets of test conditions with their respective controls (Table 1). The parameters obtained for these test conditions, e.g., growth profile, maximum specific growth rate (µmax) and cell concentration, were compared with parental control cells (Fig. 2 and Table 1). The recombinant protein expression profile and end-product formation, when hosts were subjected to different media and induction conditions, were also obtained (Fig. 2) to comprehend the dynamics of the protein expression profile, which can then be correlated with the above-mentioned parameters.

**Table 1:** Microbial growth characteristics under different media and induction conditions.

*E. coli* Strain	Growth Medium	Induction time (hour of cultivation)	Type of strains	Max Specific growth rates (μ_max_)	Cell concentration (Dry Cell wt/l)
*E.coli* M15	Defined (M9)	Early (0 h)	Control	0.38	2.21
			Test	0.30	2.00
		Mid (4.5 h)	Control	0.44	2.22
			Test	0.42	2.65
	Complex (LB)	Early (0 h)	Control	1.04	1.36
			Test	0.84	2.08
		Mid (2.5 h)	Control	1.09	1.39
			Test	1.07	2.13
*E.coli* DH5⍺	Defined (M9)	Early (0 h)	Control	0.28	2.48
			Test	0.27	2.32
		Mid (6 h)	Control	0.32	2.28
			Test	0.37	3.85
	Complex (LB)	Early (0)	Control	0.41	1.28
			Test	0.43	2.24
		Mid (3 h)	Control	0.57	1.37
			Test	0.49	2.28

We have observed from the growth profiles of all the cells (Fig. 2a1–a4) that the µmax of host cells cultured in the M9 medium was lower as compared to the LB medium (Table 1), irrespective of the host strain used. The µmax was ~ 3- and 1.5-fold lower for *E. coli* M15 and DH5α strains, respectively, when grown in the defined medium as compared to the complex medium (Table 1). In both types of strains, the cell titer (dry cell weight per liter) obtained was relatively higher in the defined medium as compared to the complex medium (Table 1). An exception to this observation was one condition for the test sample where the early growth phase titer of *E. coli* M15 was slightly higher in complex medium. Moreover, a noticeable lag phase was observed in the growth profile of cells grown in the defined medium (Fig. 2a1,a3), which was absent when cells were cultivated using the complex medium (Fig. 2a2,a4). A delay in the attainment of stationary phase, whether grown in defined or complex media, has also been observed in the case of *E. coli* M15 cells expressing recombinant protein (Fig. 2a1) as compared to the control (Fig. 2a2). The effect of protein induction at different phases of cell growth (early at OD_600_ ~ 0.1 and mid-log at OD_600_ ~ 0.6) was also observed on cell growth. Induction at the mid-log phase resulted in a higher growth rate, irrespective of the strains and the media used (Table 1). It was observed earlier that substrate and nutrient utilisation by the host plays an important role in determining the growth and, consequently, the death rate of cells^20^, with slower growth leading to an exponentially slower death.

We further analysed the recombinant protein expression profile of different cultures on SDS-PAGE gel by loading equal quantities of protein extract (i.e., 50 µg) (Fig. 2b). The samples were taken at two time points: mid-log phase of growth, i.e., OD_600_ of ~ 0.8, and late log phase of growth, i.e., 12 h of post-inoculation. The expected molecular mass of AAR is 37.65 kDa, and thus a protein band near to this size in the test samples that is absent in the control samples would be an indication of induction of the recombinant AAR in the host. As expected, induction at the early phase of growth led to an early expression of recombinant protein in both the host strains cultured in different media (Fig. 2b1,b3); however, this expression eventually diminished in the late growth phase of 12 h, especially when cultured in M9 medium (Fig. 2b2,b4). Induction at the mid-log phase retained the expression level of the protein even during the late growth phase (Fig. 2b1–b4). Protein expression comparison uncovers dynamic patterns influenced by both induction timing and variations in culture media. Inducing expression during the early growth phase indeed triggers rapid protein production across all cultures, reflecting the heightened metabolic activity characteristic of this stage. However, the subsequent decline in expression during the late growth phase, particularly evident in the M9 medium, suggests underlying complexities. This reduction may stem from resource depletion or metabolic changes as cells approach the stationary phase. In contrast, induction during the mid-log phase presents an intriguing alternative, maintaining steady protein expression levels throughout the growth phases. This sustained expression underscores the favorable physiological conditions during the mid-log phase, likely optimized cellular resources for protein synthesis. Moreover, the differential impact of culture media on protein expression dynamics reveals subtle nuances. While both media initially support expression, the more pronounced decline in the M9 medium hints at its limited capacity for sustained protein production, possibly due to a lack of essential nutrients or metabolic intermediates crucial for synthesis. Overall, this comprehensive analysis highlights the intricate interplay between induction timing, growth phase dynamics, and culture media composition, offering valuable insights for optimizing recombinant protein expression strategies.

We also tested the functional integrity of the expressed recombinant protein by analysing the product of an AAR-catalyzed reaction, i.e., hexadecanal, which is eventually converted into hexadecanol with the help of endogenously expressed aldehyde reductase enzyme^21^. The product formation was monitored by GC–MS analysis^22^, and in all the conditions, the respective maximum yield was obtained in the late log or stationary phase of the growth curve (Fig. 2c). Strain *E. coli* M15 yielded a higher titer of hexadecanol as compared to *E. coli* DH5α independent of the media used (Fig. 2c1–c4). Moreover, induction at the mid-log phase (OD_600_ of 0.6) has yielded higher product titer in both the strains and the media as compared to early phase induction (Fig. 2c1–c4). Between the two media tested, interestingly, the defined M9 medium yielded a higher product (Fig. 2c1–c4). In this medium, the protein expression in *E. coli* DH5α was significantly less than *E. coli* M15 for mid-log induction (Fig. 2b), which could be the reason for lesser hexadecanol production. However, low-level production of hexadecanol in *E. coli* DH5α in LB medium despite reasonable AAR expression during mid-log induction pointed out host-specific factors assisting in catalysis and export of hexadecanol.

### Insights into differentially expressed host cell proteins upon recombinant protein production

After elucidating the parameters to obtain optimum expression levels of functional recombinant protein in a host, we attempted to gain insights into the dynamics of the host’s proteome profile by using HRMS (high-resolution mass spectrometry) ^23,24^. We only considered the mid-log (OD_600_ of ~ 0.6) induction condition for our proteomic study as it yielded the highest recombinant enzyme and its catalytic product (Fig. 2b,c). A combinatorial proteomic study of post-induction cultures of *E. coli* M15 and *E. coli* DH5⍺ strains expressing rAAR cultivated in LB or M9 medium was conducted and compared with the control condition. Usage of both complex and defined media helped to comprehend carbon substrate usage and cellular dynamics in its metabolic network. M9 medium was supplemented with a known concentration of carbon substrate (2% glucose) as well as growth nutrients that aid metabolism, while LB medium was utilized as a rich source of the carbon substrate. We focused on two stages of growth, the post-induction mid-log and late-log phases of growth, to better understand the mechanism and cellular alterations upon recombinant protein production.

The bottom-up proteomics approach was used to compare the host cell (control) and recombinant strain (test) (data available on ProteomeXchange, online database). These samples were analysed in duplicates according to the technique outlined in the methods section and validated at a 1% false discovery rate (FDR). The number of proteins detected in *E. coli* M15 (862 to 1192) (Fig. S1a1–a4) was less than *E. coli* DH5α (1206 to 1635) (Fig. S1b1–b4, Supplementary Sheet 3). In addition, as expected, the number of proteins detected in the cells cultivated in the M9 medium was greater than those cultivated in the LB medium, as cells have to synthesise many additional sets of cellular machinery to grow in the defined medium. Moreover, both strains synthesized a distinct set of proteins during the mid-log growth phase compared to the late-log growth phase, as illustrated in Fig. S1.

**Figure 3:**
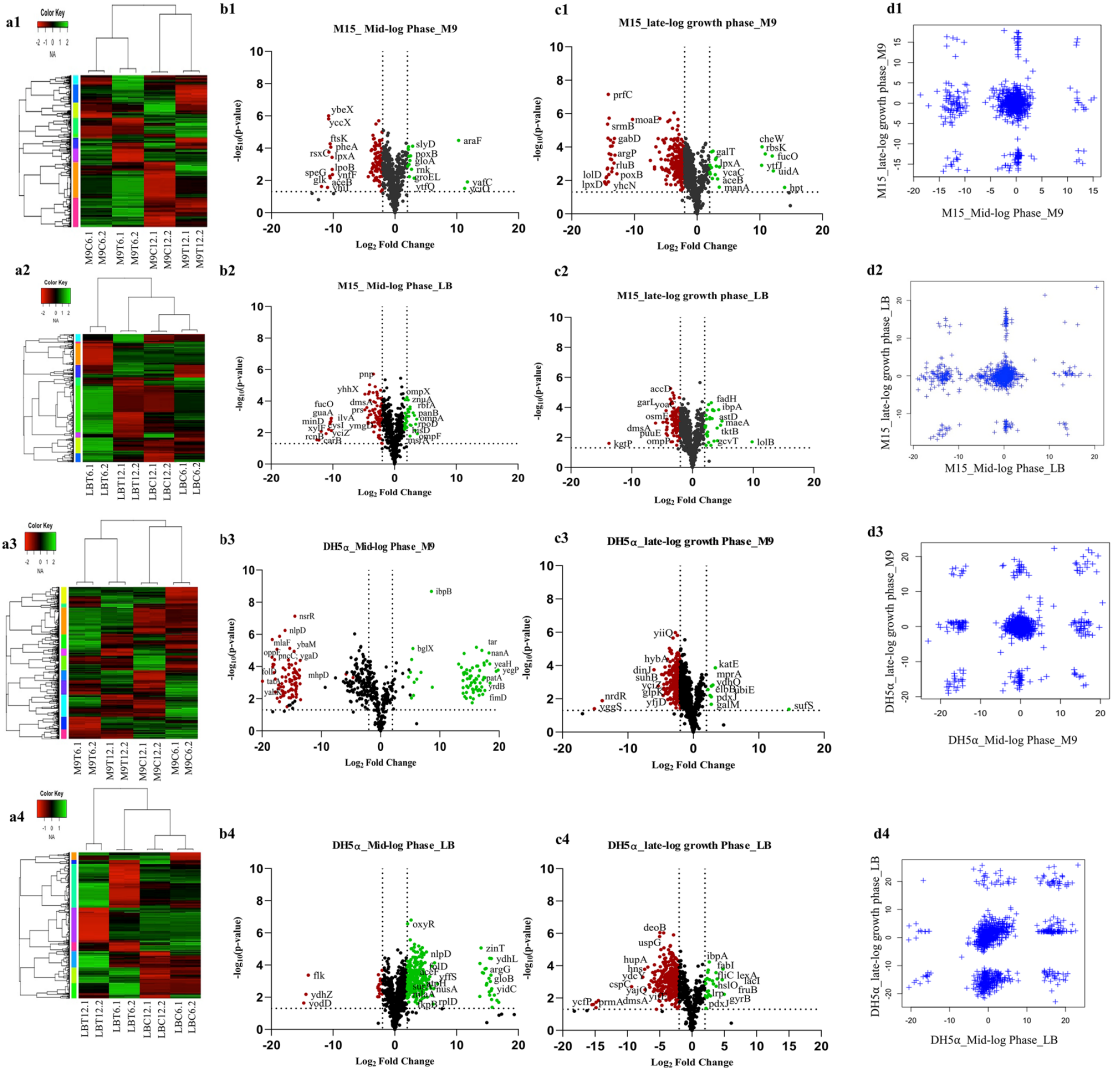
Differential gene expression analysis of recombinant protein-expressing cells with that of control. Row ‘First’ (i.e., **a1**, **b1**, **c1** and **d1**) represents *E. coli* M15 grown in M9, Row ‘Second’ (i.e., **a2**, **b2**, **c2** and **d2**) represents *E. coli* M15 grown in LB, Row ‘Third’ (i.e., **a3**, **b3**, **c3** and **d3**) represents *E. coli* DH5⍺ grown in M9, and Row ‘Fourth’ (i.e., **a4**, **b4**, **c4** and **d4**) represents *E. coli* DH5⍺ grown in LB. Heat maps, denoted as a1, a2, a3, and a4, illustrate the growth of two strains of E. coli (M15 and DH5⍺) in M9 and LB media during mid-log and late-log phases. Volcano plots, labelled **b1**, **b2**, **b3**, and **b4** for mid-log phase, and **c1**, **c2**, **c3**, and **c4** for late-log phase, demonstrate the differential expression of genes post-induction in *E. coli* M15 and DH5⍺ strains grown in LB and M9 media. The Scattered plots, represented by **d1**, **d2**, **d3**, and **d4**, depict the correlation matrix of differentially expressed proteins in mid-log and late-log growth phases of E. coli M15 and DH5⍺ strains in LB and M9 media.

The resulting proteomic data was further analysed, and the proteins with a P-value of less than 0.05 and a fold change (log2) of more than ± 2 of recombinant samples with respect to control were statistically shortlisted using R programming (Materials and Methods section), as shown in Fig. 3 and the Supplementary Sheet 1. Hierarchical clustering by differentially expressed genes is shown by heat map (Fig. 3) with substantial independent clusters (Fig. 3a1–a4), which indicated a noticeable difference between the control and test (recombinant) samples. The mid-log (the samples labelled as ‘− 6.1 and − 6.2 in Fig. 3a) and late-log phase (the sample labelled as ‘− 12.1 and − 12.2 in Fig. 3a) samples of all the control strains clustered together while the test samples either formed a separate cluster (Fig. 3a2,a4 or the same cluster (Fig. 3a3). However, a deviation was observed for the *E. coli* M15 strain cultured in M9 medium (Fig. 3a1), where control and test data clustered together for the mid- and late-log extracted samples, respectively. This indicates that the *E. coli* M15 cells resist the change in cellular protein expression dynamics upon recombinant protein expression throughout the growth phase. Volcano plots, as shown in Fig. 3b1–b4,c1–c4, were generated to observe differentially expressed proteins (DEPs). Significant differences in the host-cell-protein dynamics could be clearly observed in the host cells producing rAAR in different media as compared to the control. The range of fold change in the expression of host proteins due to recombinant protein production was similar in LB for both *E. coli* M15 and *E. coli* DH5⍺ cells (+ 20.5 to -19.0 and + 23.5 to -17.8 for mid- and late-log phase of growth, respectively, for M15; + 23.2 to -20.6 and + 25.8 to -22.8 for mid- and late-log phase of growth, respectively, for DH5⍺). However, *E. coli* M15 showed less drastic change in M9 as compared to *E. coli* DH5⍺ (+ 15.2 to − 18.7 and + 17.9 to − 16.7, respectively, for mid- and late-log phase of growth, respectively, for *E. coli* M15; + 20.5 to − 23.1 and + 22.2 to − 18.3, respectively, for *E. coli* DH5⍺ (Supplementary Sheet 1). An overall trend in number of differentially expressed host cell proteins (HCP) was analysed in all conditions, and it was found that the numbers were higher in the mid-log phase of growth as compared to the late-log phase of growth (Fig. 3b1–b4. Interestingly, in all the conditions except *E. coli* DH5α in LB, more cellular proteins got downregulated than upregulated when AAR was recombinantly expressed (Fig. S2). Scatter plots (Fig. 3d1–d4) were generated to identify unique groups of differentially expressed proteins (Supplementary Sheet 1). The data generated were further applied to look for pathway enrichment analysis using PANTHER version 14^25^, KEGG^26^ and Biocyc^27^ online modules.

### Metabolic mapping: growth and recombinant protein expression is enhanced through metabolism

The simple identification and quantification of proteins from a cell proteome is insufficient for a complete comprehension of complicated biological mechanisms. The differences in the growth profiles (Fig. 2) are indicative of the variable-activated metabolic pathways when host cells expressing rAAR were subjected to different growth conditions (A-H in Table 1). When cells were grown in an LB medium, the growth rate was very fast without lag phase (Fig. 2a2,a4, Table 1), which suggested that there would be more active metabolism involved in biomass formation. On the other hand, growth in the M9 medium (Fig. 2a1,a3, Table 1) was slower as compared with the LB medium with proper metabolism to form higher products at the stationary phase. Intriguingly, the results show that an early-stage (OD_600_ ~ 0.1 induction) formation of recombinant protein doesn’t help to produce an optimum product, but slower growth and mid-log induction (OD_600_ ~ 0.6 induction) of recombinant protein helps to make maximum product yield (Fig. 2c1–c4).

**Figure 4:**
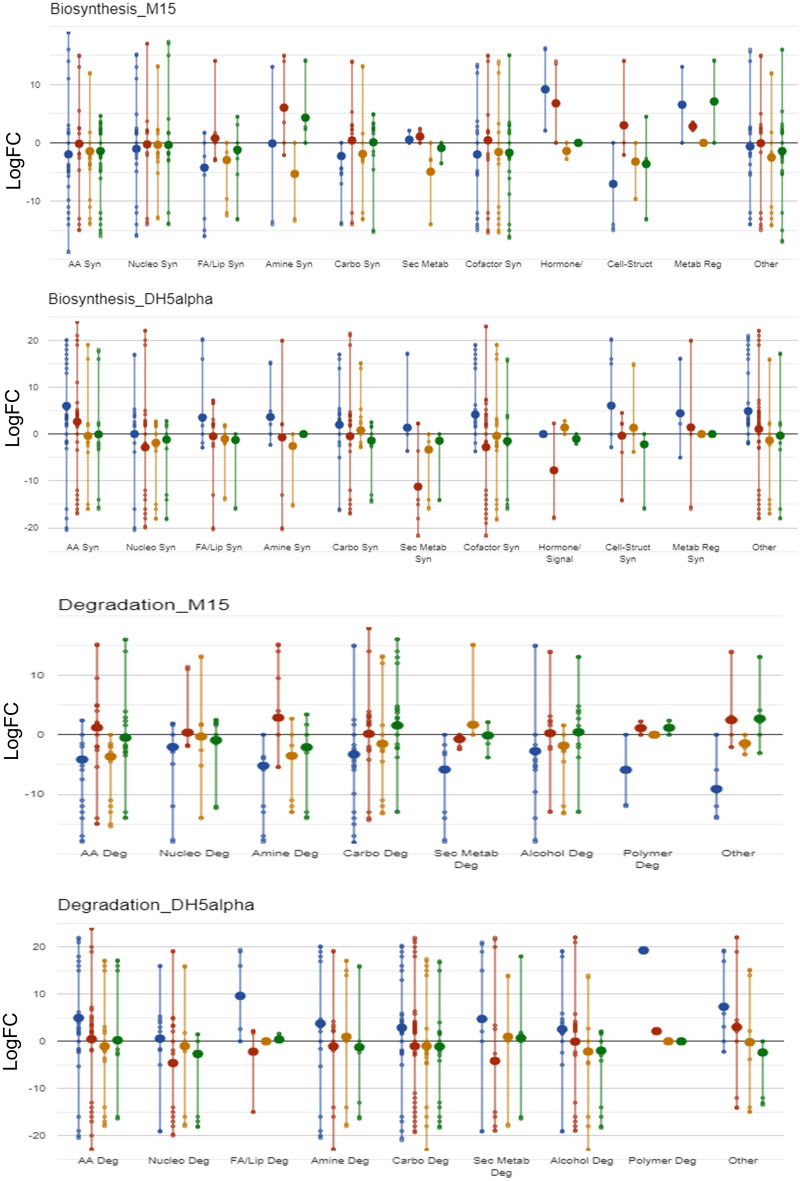
Cellular metabolic mapping in *E. coli* M15 and *E. coli* DH5⍺ strain. Colour coding—LB: mid-log phase—Blue & late-log phase—Red; M9: mid-log phase—Yellow & late-log phase—Green. The data indicates upregulation or downregulations of proteins involved in the biosynthetic or degradation pathways of cellular metabolism. Abbreviations: AA Syn—Amino acid synthesis; Nucleo Syn—Nucleotide synthesis; FA/Lip Syn—Fatty acid and lipid synthesis; Amine Syn—Amine synthesis; Carbo Syn—Carbohydrate synthesis; Sec Metab—Secondary metabolite synthesis; cell-struct-cell structure; Metab Reg—Metabolic regulation.

**Figure 5:**
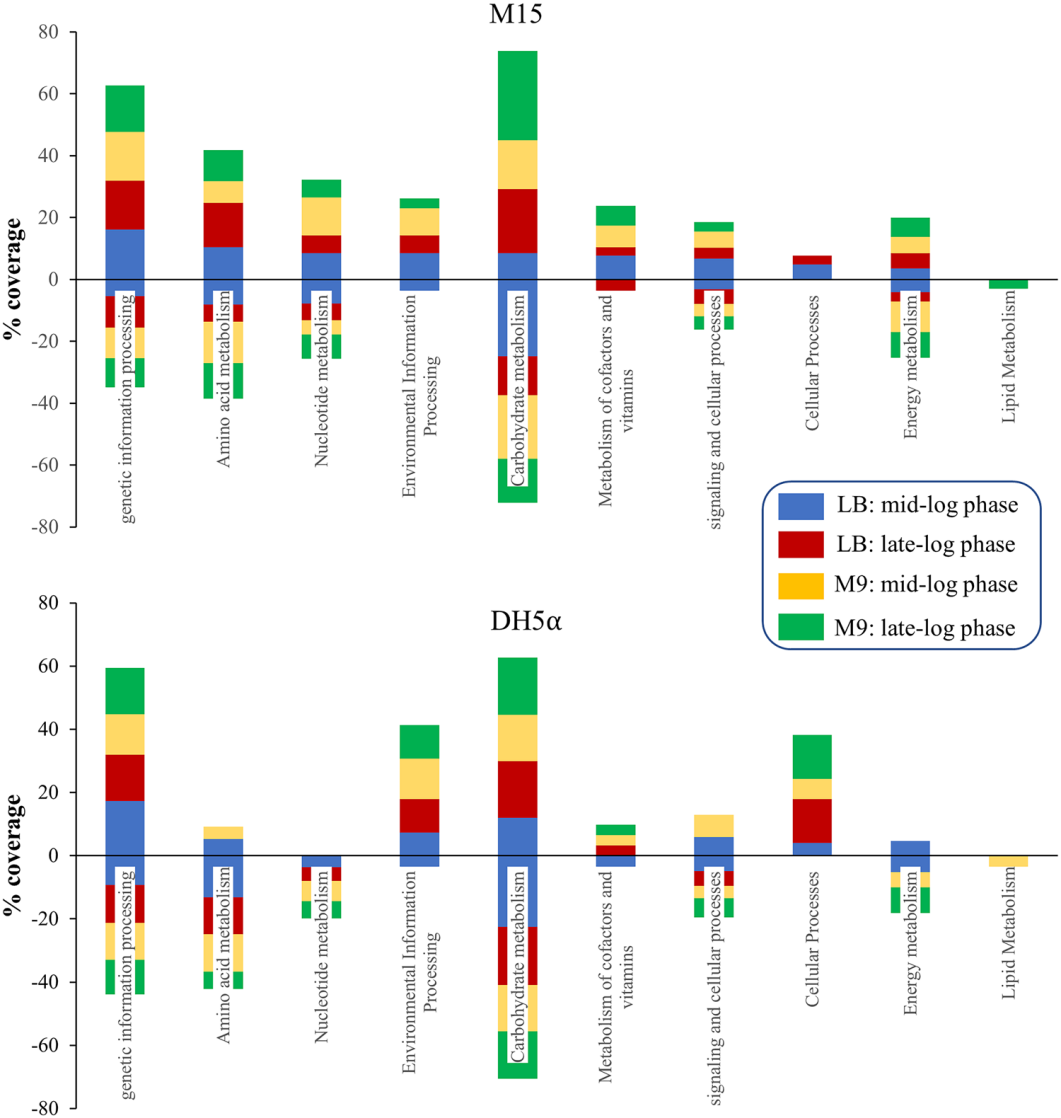
KEGG mapper of different conditions in *E. coli* M15 and *E. coli* DH5α hosts during mid-log and late-log growth phases. The Y-axis represents the percentage coverage of KEGG pathways for the proteins expressed under different growth conditions. The X-axis represents various KEGG pathways detected in the analyzed cellular proteins.

Therefore, optimized growth conditions and nutrient environment aid in achieving a high yield of recombinant protein and, in turn, product. The highest recombinant protein and product yield was obtained when *E. coli* M15 cells in the M9 medium were induced during the mid-phase of growth (Table 1, Fig. 2b2,c1). Thus, understanding the metabolism during both mid-log and late-log growth phases of all the conditions, especially the highest yielding condition (i.e., *E. coli* M15 cells induced during the mid-phase of growth in the M9 medium) and its comparison with other sets, will infer a deeper understanding of the phenotypic and genotypic differences among different hosts in use. To integrate protein expression data into metabolic data, we have used the online bioinformatic tool BioCyc with a host-specific database (*E. coli* M15 and *E. coli* DH1 (the parental strain of *E. coli* DH5α)). It shows the overlapping of genetic differences with respect to the metabolic system (metabolic mapping). The term cell map or structural proteomics refers to proteomics research whose purpose is to map out the structure of protein complexes or proteins present in a specific cellular organelle ^28^. Cellular metabolism was obtained using a KEGG Mapper under high-confidence statistical conditions, and the obtained gene information was used to conduct metabolomic mapping on the BioCyc platform, leading to information on synthesis, degradation, energy, cellular process, central dogma, stimulus–response, and cell exterior metabolism (Figs. 4, S3). After observing a significant set of impacted cellular protein expression upon heterologous protein production (Fig. 4), their annotation was carried out using PANTHER version 14 (Fig. S5 and supplementary Sheet 2), and KEGG mapper as represented in Fig. 5. Functional analysis revealed enrichment for genetic information processing, nucleotide-, amino acid-, and carbohydrate metabolism, environmental information processing, metabolism of co-factors and vitamins, signaling and cellular processes, and energy and lipid metabolism-related activities.

Amino acid metabolism had significantly changed in the condition that yielded the highest recombinant protein expression (Fig. S3). Interestingly, the dynamics in the expression profile of proteins involved in amino acid metabolism may be directly related to the composition of the amino acids in the AAR protein (non-polar amino acids (55.1%), polar amino acids (21.8%), and basic amino acids (11.1%)), especially in condition that yielded the highest recombinant protein. Enzymes involved in biosynthesis and degradation of amino acids were detected, and the levels were found to be significantly upregulated and downregulated, respectively, in the recombinant host for the prevalent amino acids (Ala, Gly, Ile, Glu, Thr, Arg) in rAAR protein. *gcvT* encoding aminomethyltransferase GcvT (Gly); *astB* and *astD* encoding N-succinylarginine dihydrolase AstB and aldehyde dehydrogenase AstD involved in synthesis of glutamate from arginine (Glu); *thrC* encoding threonine synthase (ThrC) (Thr); *argD*, *argE*, *argB* (Arg), *pheA* (Phe), *dapF* (Lys), *proA* (Pro), *metC* (Met), *trpC* (Trp), *tnaA* (Trp) involved in biosynthetic pathways of the respective amino acids (shown in bracket) were found to be significantly upregulated in the highest yielding condition. Moreover, levels of enzymes involved in degradation pathways of predominant amino acids were detected to be present in lower levels in condition (i.e., *E. coli* M15 cells induced during the mid-phase of growth in the M9 medium). D-amino acid dehydrogenase (DadA) enzyme encoded by *dadA* (Ala); Acetolactate synthase and threonine deaminase encoded by *ilvA* and *ilvI* (Thr) were also detected in lower levels in the recombinants as compared to the control. Another enzyme involved in the racemization of alanine encoded by the *dadX* gene was found to be upregulated (~ 16 fold) in the highest yielding condition (Supplementary Sheet 1).

Intriguingly, the less prevalent amino acids in rAAR followed the opposite trend. It was observed that the biosynthetic pathways of non-abundant amino acids in a recombinant protein, which in the present case includes Cys, His, Trp, and Tyr were upregulated in conditions other than the highest yielding strain (Fig. S3). Whereas diminished or no change for the enzyme dynamics profile of scarcely present amino acids was observed for the highest yielding condition except for that of Trp biosynthetic pathway. For instance, the enzymes involved in biosynthetic pathways of Cys (*cysE*), His (*hisH*, *hisF*), and Tyr (*tyrB*) were remarkably upregulated in the recombinant DH5⍺ strains with no change in the highest yielding M15 strain.

**Figure 6:**
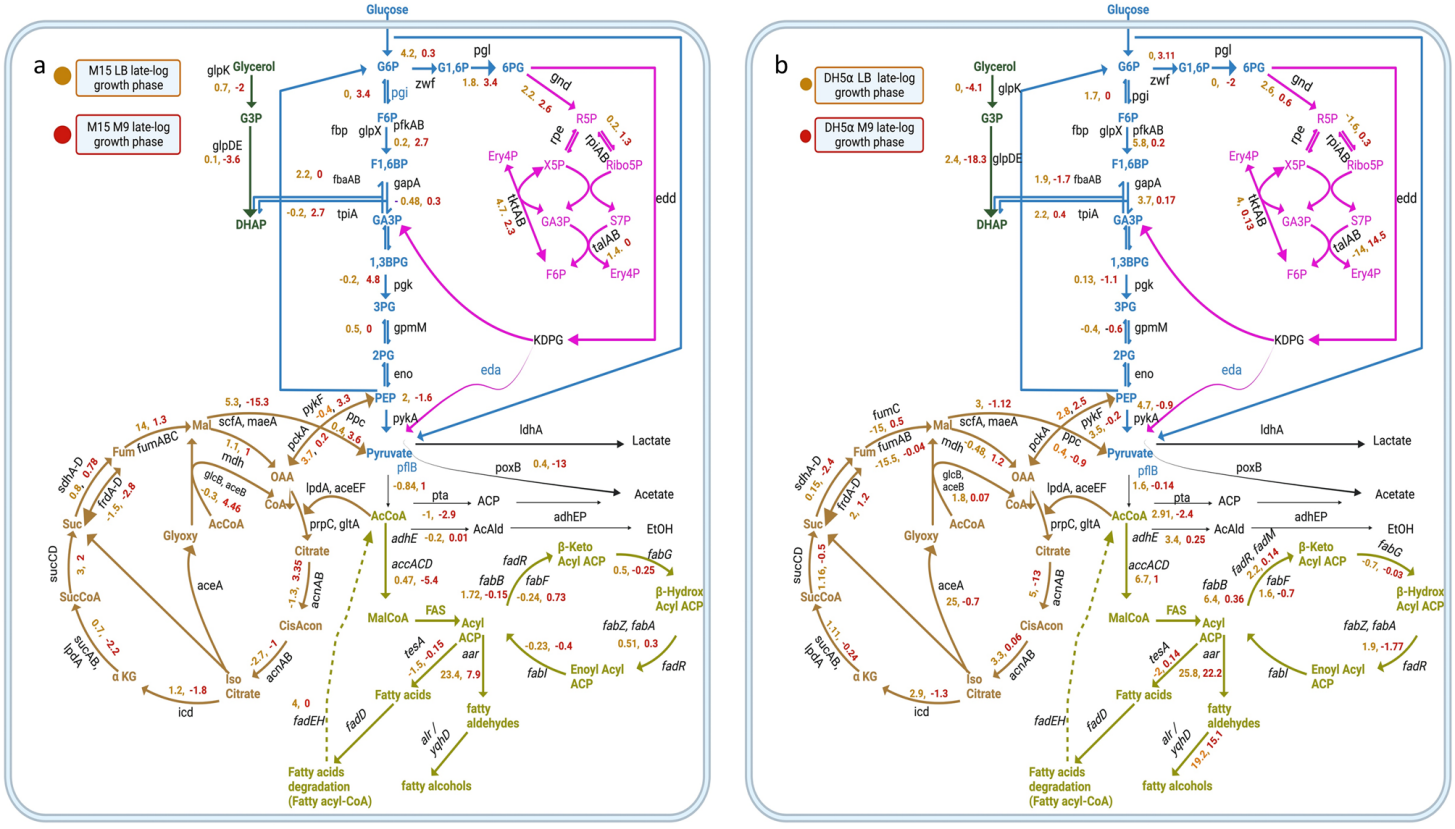
Insights of metabolic pathways that are modulated during recombinant protein production in *E. coli* M15 (**a**) and *E. coli* DH5⍺ **(b)**. Colour coding are as follows—Blue: Glycolysis, Purple: Pentose phosphate pathway, Brown: Citric acid cycle, and Yellow: Fatty acid biosynthesis pathway. Numbers indicate fold change values of recombinant strain with respect to control. Raw data has been provided in Supplementary Sheet 3.

The lipid metabolism is typically regulated via its precursor fatty acyl CoA. This precursor is also one of the substrates for AAR, and enzymes synthesizing/utilizing this precursor were upregulated/downregulated, respectively, in the highest yielding condition out of all the conditions. *dhaL* gene encoding dihydroxyacetone kinase subunit L is downregulated by ~ 13-fold in condition (i.e., *E. coli* M15 cells induced during the mid-phase of growth in the M9 medium) as compared to the control (Fig. 4, Supplementary Sheet 1). The suppression of the gene results in diminished glycerol degradation resulting in driving glycerol-driven fatty acyl CoA synthesis pathway in the highest yielding condition. *yciA* gene encoding acyl-CoA thioesterase protein was found to be significantly downregulated (~ 16.5 fold) in case of the highest yielding condition. The protein is responsible for hydrolysing coenzyme A moiety from fatty acyl-CoA. The downregulation of the *yciA* gene product thereby results in increased availability of the substrate (fatty acyl CoA) for the produced AAR protein and, in turn, results in increased product formation. Recombinant protein production manipulates cellular metabolism (Figs. 6a,b, S6) to be driven towards substrate accumulation, which in the present case is fatty acyl-ACP/CoA ^29,30^.

The genetic information processing proteins, including dysregulation of transcriptional and translational proteins (Supplementary Sheet 1), can compromise protein yield, quality, and cell viability. We observed two prominent changes in the list of transcriptional regulatory proteins within the highest yielding condition, i.e., *E. coli* M15 grown in M9 medium and induced at mid-log phase, which were absent in other conditions. The expression of *yafC* was upregulated by 15-fold, while *rcsB* was downregulated by ~ 15-fold (Fig. S4). The YafC is predicted to be a transcription factor that regulates genes related to metabolism and chlorine resistance^31^, and RcsB is a response regulator involved in biofilm and exopolysaccharide formation^32^. The changes in these two transcriptional regulatory proteins, i.e., upregulation of *yafC* and downregulation of *rcsB*, certainly seem to have a favourable impact on rAAR expression. The peptide deformylase (Pdf) enzyme encoded by the *def* gene was found to be significantly upregulated in the highest yielding condition and all the other conditions *E. coli* M15 cells were subjected to, which was not the case for *E. coli* DH5⍺ cells (Fig. S4). Intriguingly, Pdf is responsible for assuring kinetic control of nascent protein biogenesis by acting as an early chaperone. This helps in enhancing polypeptide formation and, in turn, recombinant protein expression^33,34^. It can be claimed that cellular conditions offered by M15 cells are translationally conducive, resulting in heightened functional recombinant protein production as compared to *E. coli* DH5⍺ cells, which is reflected in terms of product (hexadecanol) formation as well. A very fascinating observation had been made out of the given data in the highest yielding condition. A significant downregulation of the tRNA ligases (TyrS, TrpS, HisS, CysS) of the respective bottom-most abundant amino acids (Tyr, Trp, His, Cys) present in the AAR protein was observed (Fig. S4). However, for amino acids of intermediate abundance (Arg, Glu, Thr (Supplementary Sheet 1)), an increased expression in the respective tRNA ligases (ArgS, GltX, Thr) were detected. An unexpected slight downregulation of the tRNA ligases (AlaS, LeuS) for the topmost abundant amino acids (Ala, Leu) of AAR was also noticed as a result of auto-repression^35^. Such a comprehensive balance in terms of various tRNA ligase expression levels on the basis of amino acid composition had been observed only in the highest yielding condition out of all the conditions cells were subjected to recombinant AAR production.

Changes in regulators of carbohydrate metabolic pathway genes were also observed. In another condition, proteins such as SspA and YciH are upregulated and have been shown to regulate genes involved in carbon metabolism, specifically in the tricarboxylic acid cycle (TCA cycle) and the glyoxylate shunt^36^. The glyoxylate shunt is an alternative pathway that allows bacteria to use fatty acids as a source of carbon and energy (Figs. 6a, S6). These proteins, SspA and YciH, help bacteria adapt to different nutrient conditions and optimize their energy metabolism.

The regulators of stress responses might play an important role in cell growth and recombinant protein production of transformed cells. We observed down-regulation of several genes in the highest-yielding strain that may impact cell growth and recombinant production. The downregulation of *rcsB* gene, which is a part of the Rcs phosphorelay system associated with cellular stress responses and biofilm formation whose expression negatively impacts growth in response to environmental challenges^32,37^, may be favourable to the cells. Further, reduced stress response and antibiotic resistance may occur due to the observed downregulation of *rmf* and *dinJ* ^38,39^; whereas downregulated *yciT, glnS, prfC, suhB, rluB, yfhL* can lead to inhibition of enzyme activity, accumulation of incomplete or abnormal proteins, and impact on biosynthetic pathways, potentially resulting in a decline in the growth rate of the M15 host cells^40,41^. The overexpression of specific genes, such as *zntR, yegP, hrpA, rlmN, evgA, treR, mraZ,* and *rpoS*, can potentially enhance the production of recombinant proteins in both host cells. These gene products play various roles in improving protein production and stability. For example, ZntR helps maintain zinc homeostasis, leading to improved protein folding and stability. YegP maintains membrane integrity, aiding in the secretion of recombinant proteins. HrpA acts as a chaperone for proper protein folding and prevents protein aggregation. RlmN and MraZ contribute to ribosome biogenesis, which improves protein synthesis. EvgA and TreR, which are involved in stress response, enhance cellular resources for protein production, and RpoS acts as a stress response regulator, improving protein folding and stability.

Upregulated stress response genes, like *sspA*, *rsmB*, *iscR* (for the *E. coli* M15 induced at mid-log phase), *phoQ*, *ypdF*, and *lolB*, are enhancing the ability of bacteria to adapt to changing environmental conditions. Functionally, *sspA* and *rsmB* stabilize mRNA and regulate gene expression post-transcriptionally, while *iscR* and *phoQ* regulate iron homeostasis, oxidative stress response, and membrane remodeling^42,43^. *ypdF* and *lolB* contribute to oxidative stress response, DNA replication, and outer membrane integrity^44–46^.

The expression levels of various genes in *E. coli* DH5⍺ host cells (Figs. 6b, S6b) also significantly impact recombinant protein production, including yield, quality, and efficiency.

## Discussion

This study presents a pioneering approach by leveraging proteomics to meticulously trace the intricate cellular dynamics exhibited by parental and recombinant cells under distinct growth conditions, notably in M9 defined and LB complex media, induced at strategically optimized time points. We have considered two common laboratory *E. coli* strains for this study, both being derivatives of K12 strains. One strain, i.e., *E. coli* M15(pREP4), is known to keep strict transcriptional control over the recombinant gene expression by providing extra copies of the *lacI* gene via pREP4 low-copy plasmid, thus ensuring no leaking expression. Another strain, i.e., *E. coli* DH5α, is known to have mutations in the recombinase and endonuclease genes for better plasmid stability^47,48^. When grown without heterologous protein expression (control), the parental cells showed uninterrupted growth, while recombinant cells grew at a slower rate during the growth phase in the M9 minimal medium compared to the LB medium (Fig. 2a1–a4). The optimization efforts coupled with proteomics data underscored the significance of transcription and translational machinery in contributing to the metabolic burden (Fig S4, Supplementary Sheet 1). It was evident that the metabolic burden escalated only when recombinant protein synthesis correlated with protein folding challenges during production, culminating in proteolytic degradation and ensuing cellular responses, especially heat shock reactions (Fig. S3). Notably, our findings underscore the pivotal role of the timing of recombinant protein induction within the host cell, dictating its fate (Fig. 2b1–b4). The study unveiled substantial disparities in both protein yield and product outcomes contingent upon the induction timing of recombinant protein production during different growth phases, even amidst diverse nutrient environments. Specifically investigating Acyl-(acyl carrier protein) reductase (AAR) protein synthesis in LB and M9 minimal media, our findings highlighted a significant enhancement in protein titer and product yield when inducing protein synthesis during the mid-growth phase compared to induction at the time of inoculation (Fig. 2). While the production of recombinant protein contributed to the metabolic burden, transcription or the transcript itself emerged as the primary factor (Figs. 4, 5). Furthermore, an excess of transcription or elevated levels of recombinant mRNA, in general, elicited growth retardation, albeit not necessarily severe growth inhibition, profoundly influencing metabolic product outcomes^49,50^.

The study identified a significant difference in the expression levels of proteins involved in the fatty acid and lipid biosynthesis pathway between the two host strains. Lipid metabolism, which is a pivotal cellular process responsible for the synthesis, storage, and utilization of fatty acids, appears to be strategically regulated under high recombinant protein-yielding conditions to favour the production of fatty acyl CoA (Fig. 6). In addition to fatty acyl ACP, fatty acyl CoA is also a precursor for AAR, and thus, any enhancement in its production can directly contribute to higher AAR product yield. The observed downregulation of the *dhaL* gene, which encodes the dihydroxyacetone kinase subunit L that is intrinsically linked to glycerol metabolism, in high recombinant protein-yielding *E.* coli M15 strain is significant. The downregulation of *dhaL* can lead to reduced glycerol degradation^51^. This reduction, in turn, directs the pathway towards an increase in glycerol-driven fatty acyl CoA synthesis, thereby creating a surplus of the necessary substrate for AAR production.

Furthermore, the observed downregulation of the *yciA* gene, which encodes the acyl-CoA thioesterase protein, by about 16.5-fold in recombinant *E.* coli M15 strain (Supplementary Sheet 1), is also of substantial relevance. Acyl-CoA thioesterase plays a pivotal role in lipid metabolism by hydrolyzing the coenzyme A moiety from fatty acyl-CoA^52^. The reduced expression of this protein would mean that there is a decreased hydrolysis of fatty acyl-CoA. Consequently, this leads to an accumulation of the substrate, fatty acyl CoA, creating a favourable environment for the AAR protein to function optimally, thus leading to increased product formation. The insights gathered from Fig. 6a,b, and Fig. S6 further underline the complexity of cellular metabolism and its dynamic response to recombinant protein production. Such manipulations can divert metabolic pathways to favour specific reactions and accumulate desired products. In the current scenario, the metabolic adjustments appear to favour the accumulation of fatty acyl-ACP/CoA, which serves as a crucial substrate for AAR. Overall, the observed modulation of lipid metabolism under high rAAR yielding condition, through gene regulatory mechanisms, optimally positions the cell for enhanced AAR production.

In conclusion, we examined in this study the cellular responses of *E. coli* M15 and *E. coli* DH5⍺ host cells grown under different nutrient conditions upon expression of recombinant protein. The comprehensive investigation, delving into the intricate interplay of cellular dynamics, gene regulation, and metabolic pathways, uncovers pivotal factors governing recombinant protein production. Particularly, the strategic modulation of lipid metabolism showcases a pronounced impact on AAR protein yield, elucidating the significance of pathway manipulation for enhanced product outcomes. These insights not only highlight the complexity of cellular responses to protein synthesis but also pave the way for targeted metabolic engineering strategies in microbial systems. The findings underscore the potential for optimizing bioprocessing strategies and yield enhancement by leveraging the regulatory mechanisms governing cellular metabolism, presenting a promising framework for further advancements in recombinant protein expression and desired product yield.

## Methods

### Strain use and maintenance

*Escherichia coli* strains that are derivative of K12, i.e., M15 (strain DZ291—Str^R^ F^–^ φ80*lac*ZΔM15 *thi lac*^-^ *mtl*^-^ *rec*A^+^) (Qiagen) and DH5⍺ (F^–^ φ80*lac*ZΔM15 Δ(*lac*ZYA-*arg*F) U169 *rec*A1 *end*A1 *hsd*R17 (r_K_^–^, m_K_^+^) *pho*A *sup*E44 λ^–^*thi*-1 *gyr*A96 *rel*A1) (Invitrogen), were used as host systems for the expression of a 37.5 kDa recombinant Acyl-(acyl carrier protein (ACP)) reductase (rAAR) protein. The rAAR gene was cloned in *E. coli* expression vector pQE30 with T5 promotor (Qiagen) and transformed in *E. coli* strains using the chemical transformation method. The transformed recombinant *E. coli* M15 strain is kanamycin resistant due to the presence of pREP4 plasmid (to avoid leaky expression) as well as ampicillin resistant due to the presence of pQE30 plasmid, while recombinant *E. coli* DH5⍺ is only ampicillin resistant due to the presence of pQE30-based plasmid. The strains were stored on a M9 medium containing glycerol (20%) at -80 °C.

### Medium composition and inoculum preparation

M9 modified and LB medium as described by Tabinda Shakeel et al^53^ and Sezonov, G. et al^54^ was used in this study at a glucose concentration of 2%. The pH of the medium was adjusted to 7.5 using 6N HCl and 10N NaOH solutions. For preparing inoculum, a − 80 °C glycerol stock of strain was thawed and inoculated to 50 mL medium in a 250 mL Erlenmeyer flask. The culture was incubated at 30 °C for 12 h at 150 rpm in an orbital shaker. The inoculum volume for the shake flask experiment was decided based on the initial optical density (OD) of the primary culture at 600 nm. For each run, the initial OD_600_ was kept at 0.1 for shake flask experiments.

### Shake flask experiments

All shake flask experiments were performed in 2000 mL baffled Erlenmeyer flasks containing 100 mL of M9 minimal Medium and LB medium. For the primary culture, 50 mL medium was inoculated with glycerol stock of strain and incubated at 30 °C for 12 h at 150 rpm in an orbital shaker After 12 h, OD of the primary culture was calculated by taking absorbance at 600 nm using a spectrophotometer (SpectraMax, M1, Molecular Devices, USA). About 50 mL of M9 minimal medium and LB medium was inoculated with the primary culture where initial OD_600_ of all culture flasks were kept at 0.1. For offline measurements of cell density and metabolites (Fig. S8), 2 mL of culture broth was harvested every 2 h. At OD_600_ of 0.6, culture was induced with 1 mM final concentration of IPTG and harvested at the mid-log and late-log phase of growth.

### Cell density estimation

For cell density estimation, 2 mL of culture broth was harvested aseptically every two hours of cultivation. Cell density was calculated using dry cell weight measurement in duplicates. For dry cell weight measurement, 1 mL of cell broth was pipetted on dry 0.2 µm filter paper (Whatman, mdi) in pre-weight dry filter paper, and Filter paper was dried at room temperature and measured the post-drying weight. The mass difference was used to calculate the dry cell weight.

### Metabolite measurements

Glucose and other metabolite concentrations in the culture supernatant were determined by ion exclusion chromatography (Fig. S8). BioRad, Aminex HPX-87H column (7.8 × 300 mm, 10 μm, Cat.No: 125-0140) and HPX-87H guard column (4.6 × 300 mm, Cat No: 125-0129) were used for glucose estimation. The mobile phase consisted of 4 mM H2SO4, and a flow rate of 0.3 mL/min was used for analysis. Column temperature was kept at 40 °C. Analysis was performed using the Agilent HPLC 1200 system (Agilent Tech. Inc., USA) with a refractive index detector. For fatty alcohol analysis, an equal volume of ethyl acetate containing 10 mg/L of 1-octadecene as internal standards was added to the culture and mixed, centrifuged, and the upper organic layer was used for analysis. A gas chromatography system (GC, 7890 A from Agilent) equipped with an HP-5 column of 30 m length, 0.32 mm internal diameter and 0.25 µm film thickness and an FID detector was used for analysis. The oven programme was set as follows: initial 100 °C for 3 min, then temperature ramped up to 250 °C with rate of 10 °C/min and was then held at 250 °C for additional 10 min. The total running time of the programme was 28 min. The inlet and detector temperatures were maintained at 150 °C and 280 °C, respectively.

### Cell disruption and total protein estimation

The cells were dissolved in lysate buffer (50 mM Tris, 100 mM NaCl and 1 mM DTT at pH 7.4) and lysed using an Ultrasonicator system (VCX-150, Nugen Scientific) for 20 min at 1000 Hz for 15 s ON–OFF. After lysis, the total protein was measured in lysed supernatant using the BCA (Pierce Protein assay Kit, Thermo Scientific, USA) method and transformed AAR protein expression was identified by SDS-PAGE.

### Proteomics analysis

To prepare the samples for proteomic studies, total protein was identified by the BCA Method and 50 µg of total protein was used. 50 µg of total protein was reconstituted in 20 μL of resuspension buffer containing 7 M urea and 2 M thiourea, added 2 µl of 100 mM DTT to the solution to make the final DTT concentration 10 mM and gently vortexed and incubated at 37 °C for 30 min. This was followed by the addition of 6 μL of 100 mM iodoacetamide prepared in 30 mM and incubated at 25 °C (room temperature) in the dark for 30 min. Adjusted the pH 7–8 of the solution by addition of 25 mM ammonium bicarbonate, samples were then digested using sequencing grade trypsin (1 μg per 50 μg of total protein; Pierce Biotechnology, USA) overnight at 37 °C. The enzymatic digestion was terminated by the addition of formic acid to pH 3.0 to 4.0. The tryptic peptides were desalted using a C18 Spin column (Thermo Scientific, USA). Eluted samples were vacuum-dried and reconstituted in 0.1% (v/v) formic acid before being subjected to LC–MS/MS^55^.

### Data acquisition, processing and analysis

LC–MS/MS analysis was performed using Orbitrap Fusion Lumos Tribrid Mass Spectrometer equipped with nano-LC Easy nLC 1200 (Thermo Fischer Scientific, Singapore). Liquid chromatography separation was performed at a flow rate of 300 nl/ml on a C18 pre-column (Acclaim PepMapTM 100, 75 um X 2 cm, nanoViper, P/N 164946, Thermofisher Scientific Incorporation) followed by analytical column (Acclaim PepMapTM RSLC C18, 75um X 50 cm, 2um, 100 Å, P/N ES803). The peptides were separated using a gradient of 2% solvent B to 10% in 5 min followed by gradient increase to 45% and sharp increase to 95%, then retention of 95% for 10 min. Solvent A was aqueous solution in 0.1% formic acid, and solvent B was 95% acetonitrile in 0.1% formic acid. The eluted peptides were injected into the mass spectrometer and the MS1 data were acquired in full scan mode at 120,000 orbitrap resolution with mass range from 375 to 2000 Da. Data were acquired using the Thermo Xcalibur software setup version 4.3.73.11 (Thermo Fischer Scientific, Inc 2019). Precursors were allowed to fragment using Higher-energy C-trap dissociation (HCD) in ion trap (IT) detector with collision energy of 28 in a data dependent MSn Scan acquisition. Charge state screening of precursor ions and monoisotopic precursor selection was enabled. The parent ions once fragmented were excluded for 40 s with exclusion mass width of + /− 10 ppm. The lock mass option (polydimethylcyclosiloxane; m/z 445.120025) enabled accurate mass measurement in the MS Mode. For analysis, raw LC–MS/MS data files obtained from the mass spectrometer were processed with Proteome Discoverer™ (Version 2.4.1.15, Thermo Fisher™ Scientific Inc). The Proteome Discoverer processing workflow was employed in the label free quantitation (LFQ) of relative protein abundance across the samples and controls. For the search process, Sequest HT tools were used. Peak lists obtained from MS/MS spectra were identified using the MSF files. Protein identification was conducted against a concatenated target/decoy version of the uniport database (*E coli* + DH5⍺ + K12) and AAR peptide sequence along with Proteome Discoverer contaminant database.

The identification settings were as follows: trypsin digestion with maximum of 2 missed cleavages; minimum peptide length 6; precursor mass tolerance 10 ppm; fragment mass tolerance 0.6 Da; fixed modifications; carbamidomethyl c (+ 57.021464 Da), variable modifications; oxidation of m (+ 15.994915 Da), acetylation of protein n-term (+ 42.010565 Da). Peptides and proteins inferred from the spectrum results using Uniprot database (Escherichia + coli + DH5⍺ + K12) and AAR peptide sequence). Peptide Spectrum Matches (PSM’s), peptides and proteins were validated at a target False Discovery Rate (FDR) strict to 0.01 and relaxed to 0.05.

### Statistical analysis

The statistically significant differences between recombinant and wildtype samples were calculated using the R programme (R version 4.1.0 (2021-05-18). *P* (−log10) < 0.05 was considered as statistically significant and log2 fold of above + /− 2. All data were calculated in replicates. The code is available in the repository.

### Functional gene annotation

To find out functional gene annotation from significant proteins that have fold change greater than 2 & lesser than -2, we have used an online bioinformatics tool that is KEGG mapper assign KEGG Ortholog (KO) (https://www.kegg.jp/kegg/mapper/assign_ko.html) and PANTHER gene ontology with *Escherichia coli* (562) database ^56^, and the input sequence was sorted out using Python programming.

### Cellular metabolic mapping

Metabolic mapping was employed for significant confident proteomics data using online tool Bio Cyc, Omics viewer module (https://biocyc.org/overviewsWeb/celOv.shtml?orgid=ECOLI) with uploading mid-log and late-log phase data (Supplementary Sheet 1) with default parameters and specific host database^57^. As a result, we obtained complete metabolic pathway information, which is discussed in the result part.

### Supplementary Information

Supplementary Figures.

Supplementary Information 2.

## Data Availability

The mass spectrometry proteomics data have been deposited to the ProteomeXchange Consortium via the PRIDE^58^ partner repository with the dataset identifier PXD050495 and 10.6019/PXD050495. Statistical analysis R programming and Python code was deposited in github, reference details is https://github.com/Rish014/Proteomics.git.
