# Supplementary material for: Proteomics and metabolic burden analysis to understand the impact of recombinant protein production in E. coli
Source: Sci Rep. 2024 May 28;14:12271. doi: 10.1038/s41598-024-63148-y (PMC11133349; doi:10.1038/s41598-024-63148-y)
Supplement: Supplementary file 1 — Supplementary Figures. [file 41598_2024_63148_MOESM1_ESM.pdf]

Supporting Information

Proteomics and metabolic burden analysis to understand the impact of recombinant protein production in *E. coli*

Girish H. Rajacharya[*‡†], Ashima Sharma‡[+] and Syed Shams Yazdani‡[†]

[†] Microbial Engineering Group, International centre for genetic engineering and biotechnology (ICGEB), New Delhi, India
‡ DBT-ICGEB Centre for Advanced Bio-Energy Research, International Centre for Genetic Engineering and Biotechnology, New Delhi, India.
*School of Interdisciplinary Research (SIRe), Indian Institute of Technology, New Delhi, India

List of supporting information:

Figure S1. Venn diagrams representing differential proteins in different media (LB & M9).

Figure S2. The number of proteins differentially expressed in M15 and DH5a when recombinant AAR is expressed in LB and M9 media.

Figure S3. Overview of metabolic mapping with differential expressed genes and associated metabolism. Colour coding - LB: mid-log phase - Blue & late-log phase – Red; M9: mid-log phase - Yellow & late-log phase - Green.

Figure S4. Overview of central dogma with differential expressed genes and associated metabolism. Colour coding - LB: mid-log phase - Blue & late-log phase – Red; M9: mid-log phase - Yellow & late-log phase - Green.

Figure S5. Gene enrichment analysis of *E. coli* M15 and DH5α in LB and M9 Medium

Figure S6. Insights of metabolic pathways during recombinant protein production in *E. coli* at mid-log growth phase

Figure S7. Expression profile of recombinant protein on SDS PAGE gel.

Figure S8. Substrate and product profile of *E. coli* M15 and DH5α in M9 medium

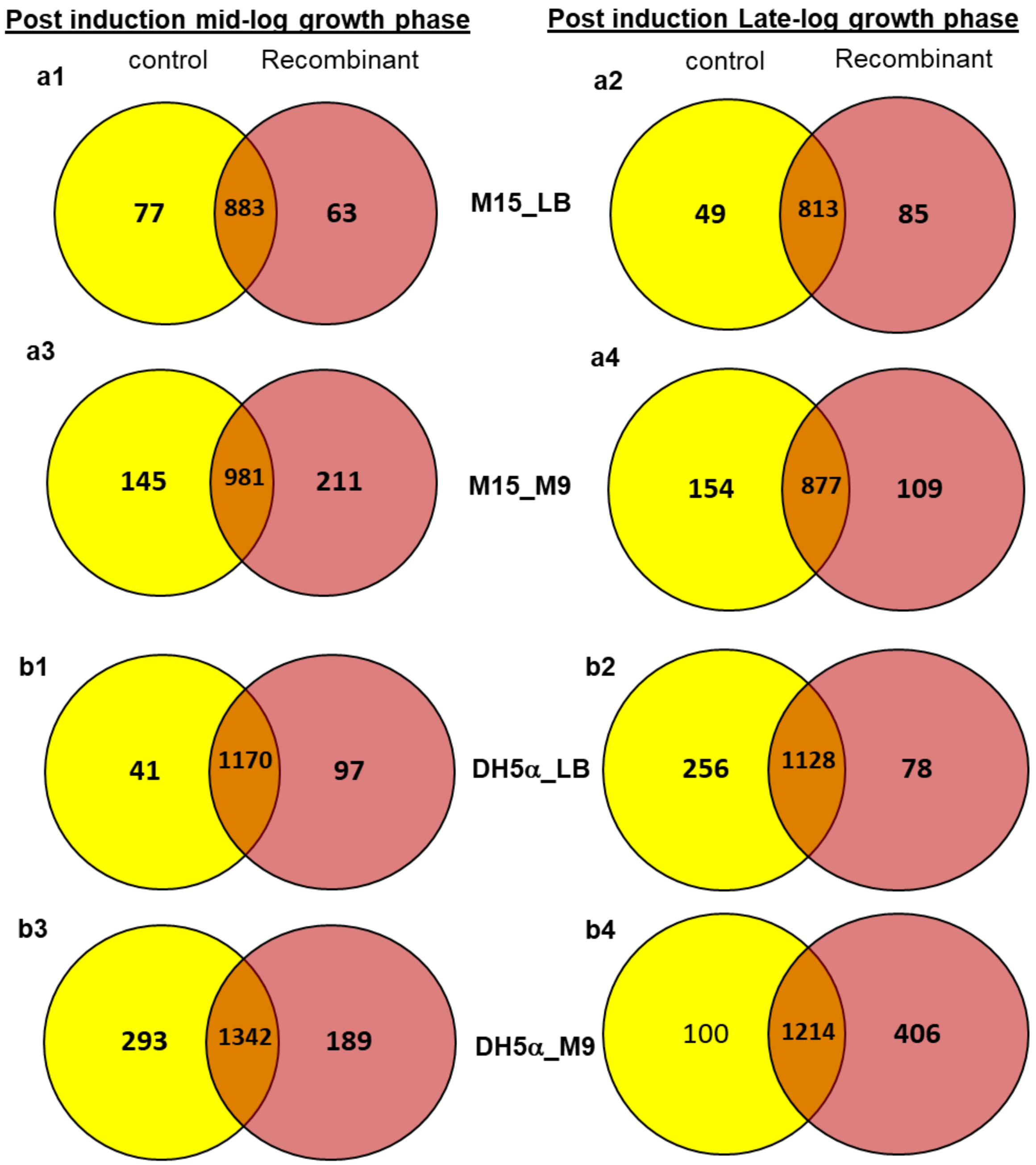

Figure S1. Venn diagrams representing differential proteins in different media (LB & M9). a1 – Venn diagram for M15_LB control vs recombinant at mid-log growth phase. a2 - Venn diagram for M15_LB control vs recombinant at late log growth phase. a3 – Venn diagram for M15_M9 control vs recombinant at mid-log growth phase. a4 - Venn diagram for M15_M9 control vs recombinant at late log growth phase. b1 – Venn diagram for DH5α_LB control vs recombinant at mid-log growth phase. b2 - Venn diagram for DH5α_LB control recombinant at late log growth phase. b3 **–** Venn diagram for DH5α_M9 control recombinant at mid-log growth phase . b4 - Venn diagram for DH5α_M9 control recombinant at late log growth phase.

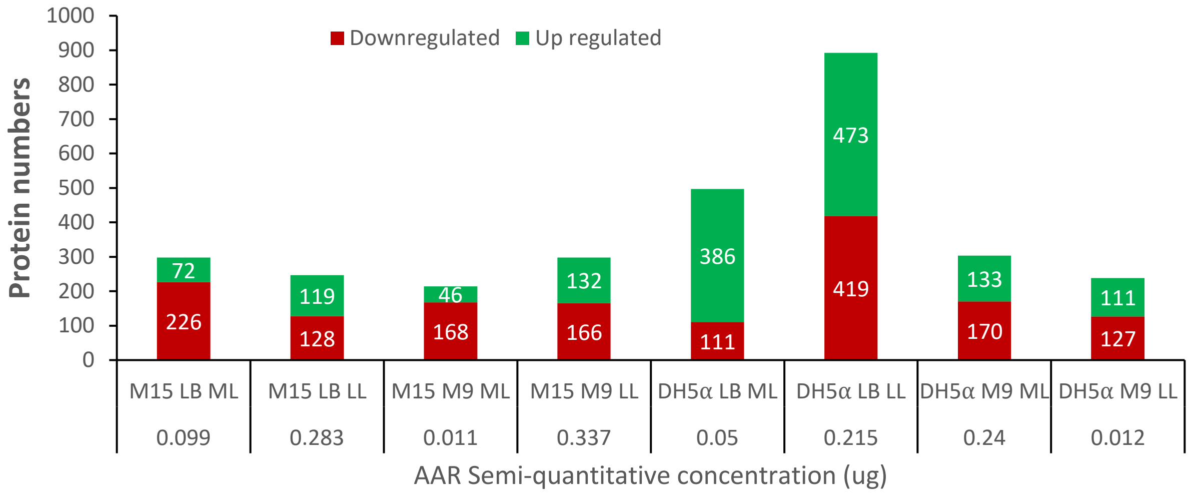

Figure S2. The number of proteins differentially expressed in M15 and DH5a when recombinant AAR is expressed in LB and M9 media. The semiquantitative value of AAR is also shown below the X-axis. Abbr: ML – Post-induction mid-log phase growth sample, LL – Post-induction late log-phase growth sample.

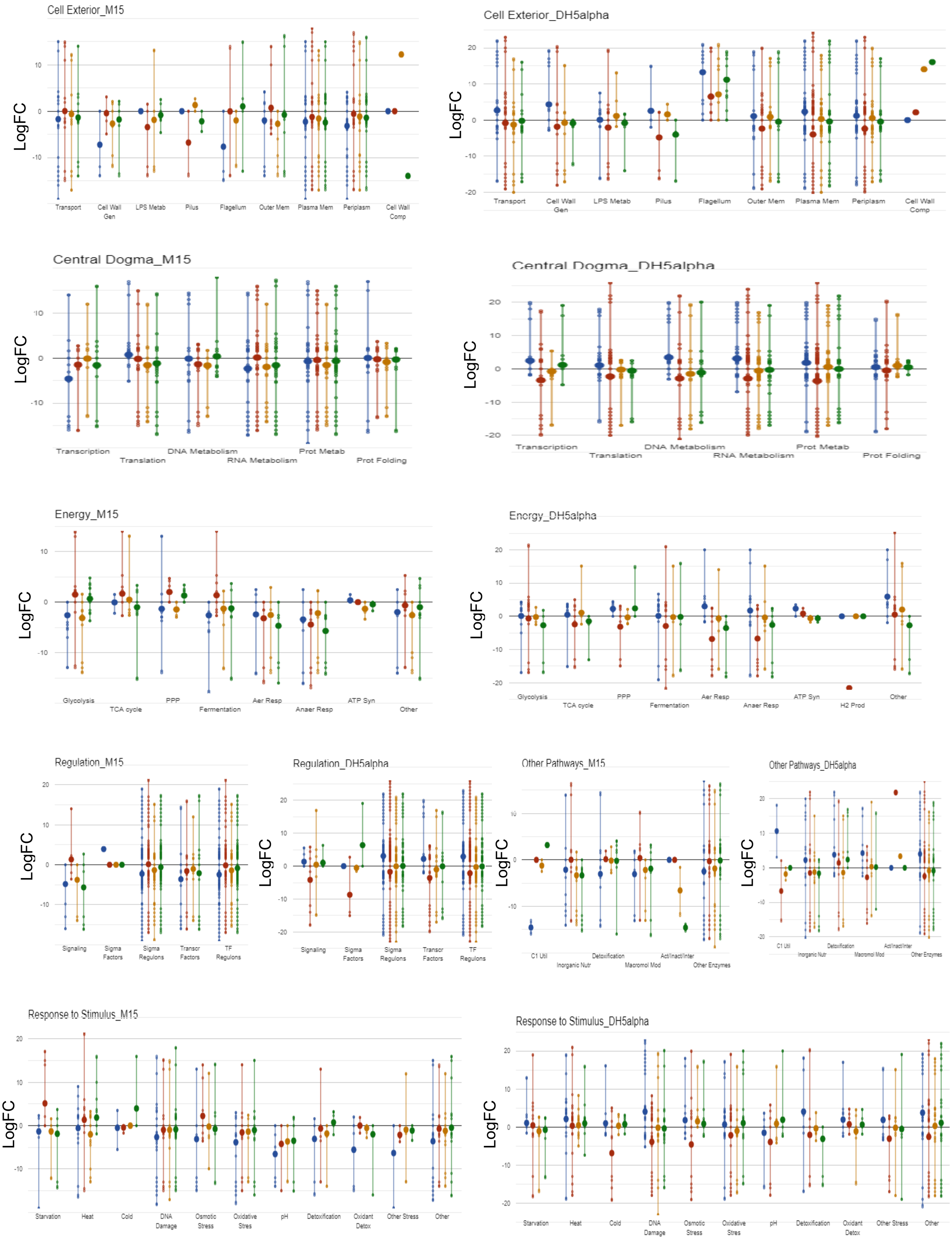

Cell Exterior_M15
LogFC
Transport
Cell Wall Gen
LPS Metab
Pilus
Flagellum
Outer Mem
Plasma Mem
Periplasm
Cell Wall Comp
Cell Exterior_DH5alpha
Central Dogma_M15
Transcription
Translation
DNA Metabolism
RNA Metabolism
Prot Metab
Prot Folding
Central Dogma_DH5alpha
Energy_M15
Glycolysis
TCA cycle
PPP
Fermentation
Aer Resp
Anaer Resp
ATP Syn
Other
Energy_DH5alpha
H2 Prod
Regulation_M15
Signaling
Sigma Factors
Sigma Regulons
Transcr Factors
TF Regulons
Regulation_DH5alpha
Other Pathways_M15
C1 Util
Inorganic Nutr
Detoxification
Macromol Mod
Act/Inact/Inter
Other Enzymes
Other Pathways_DH5alpha
Response to Stimulus_M15
Starvation
Heat
Cold
DNA Damage
Osmotic Stress
Oxidative Stres
pH
Oxidant Detox
Other Stress
Response to Stimulus_DH5alpha

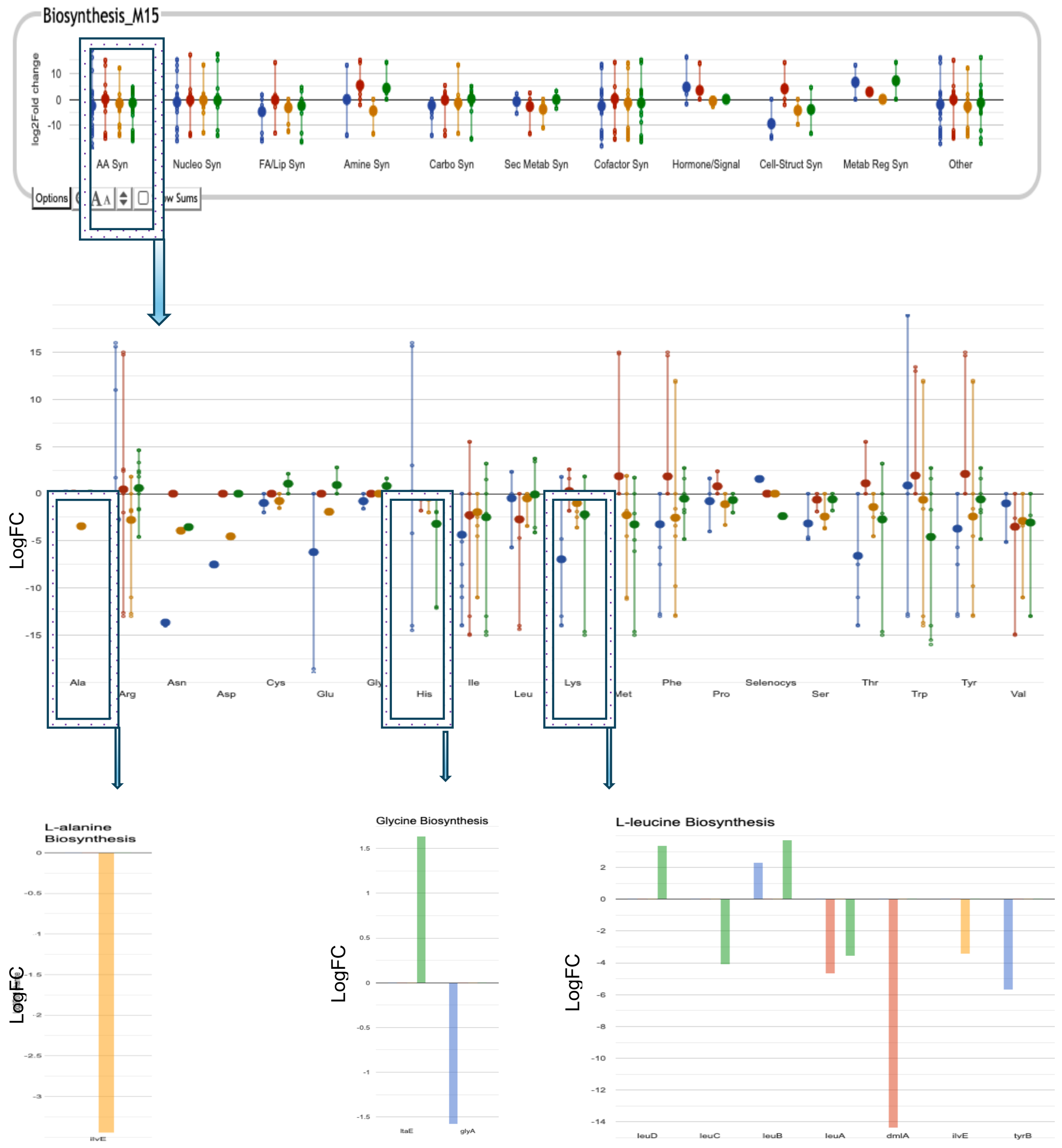
Biosynthesis_M15
log2Fold change
10
0
-10
AA Syn
Nucleo Syn
FA/Lip Syn
Amine Syn
Carbo Syn
Sec Metab Syn
Cofactor Syn
Hormone/Signal
Cell-Struct Syn
Metab Reg Syn
Other
Options
w Sums
LogFC
15
10
5
0
-5
-10
-15
Ala
Arg
Asn
Asp
Cys
Glu
Gly
His
Ile
Leu
Lys
Met
Phe
Pro
Selenocys
Ser
Thr
Trp
Tyr
Val
L-alanine Biosynthesis
ilvE
Glycine Biosynthesis
ltaE
glyA
L-leucine Biosynthesis
leuD
leuC
leuB
leuA
dmlA
ilvE
tyrB

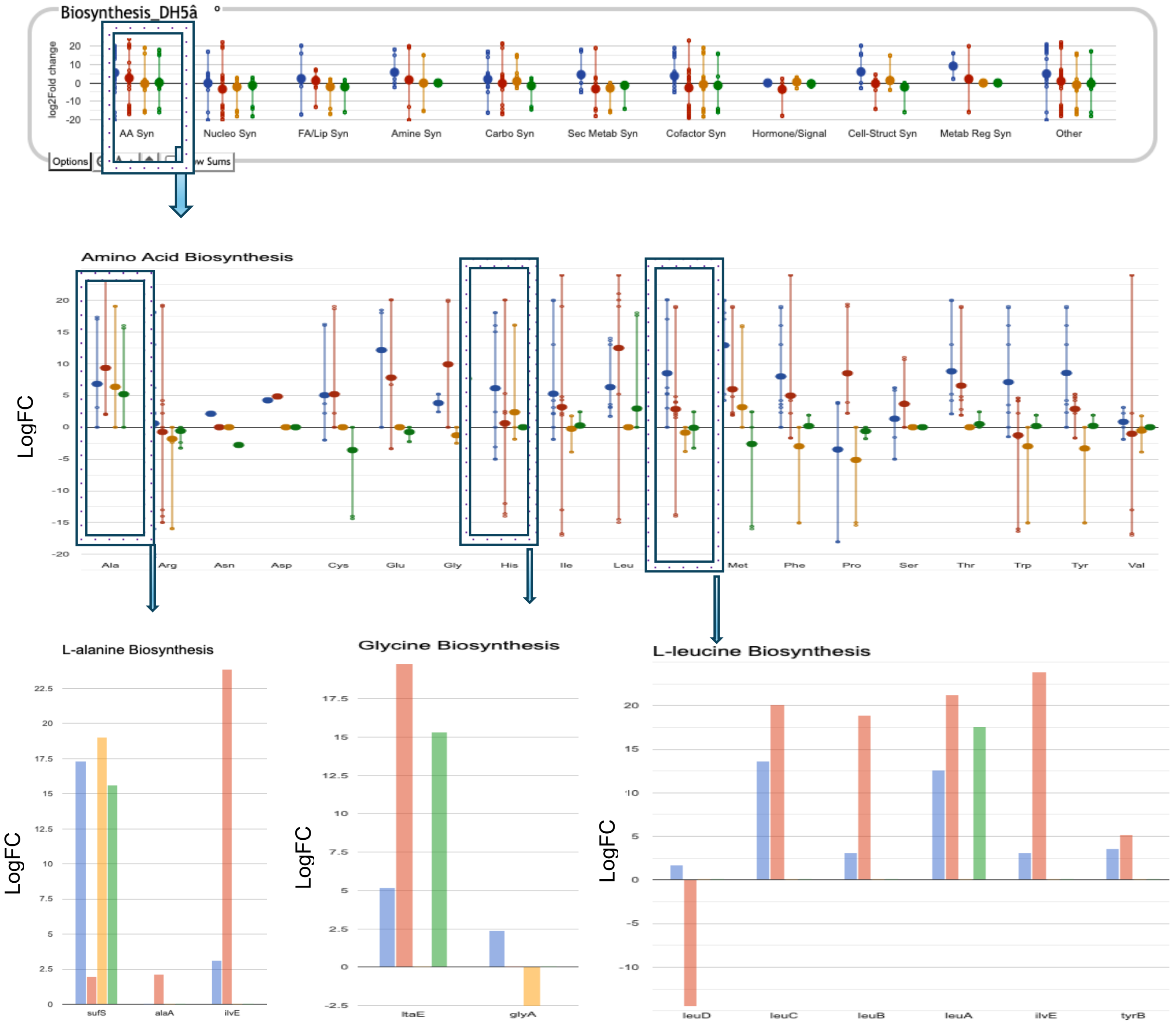

Figure S3. Overview of metabolic mapping with differential expressed genes and associated metabolism. The following link is helpful to get a detailed description – Go to https://biocyc.org/, select a specific database (here, we have selected *Escherichia coli* K-12 database, select metabolic map, add the differentially expressed genes with fold change values, select all parameters and press ok. Once we get the omics dashboard, click on an individual to get into details and differential expression along with metabolic pathways. Colour coding - LB: mid-log phase - Blue & late-log phase – Red; M9: mid-log phase - Yellow & late-log phase - Green.

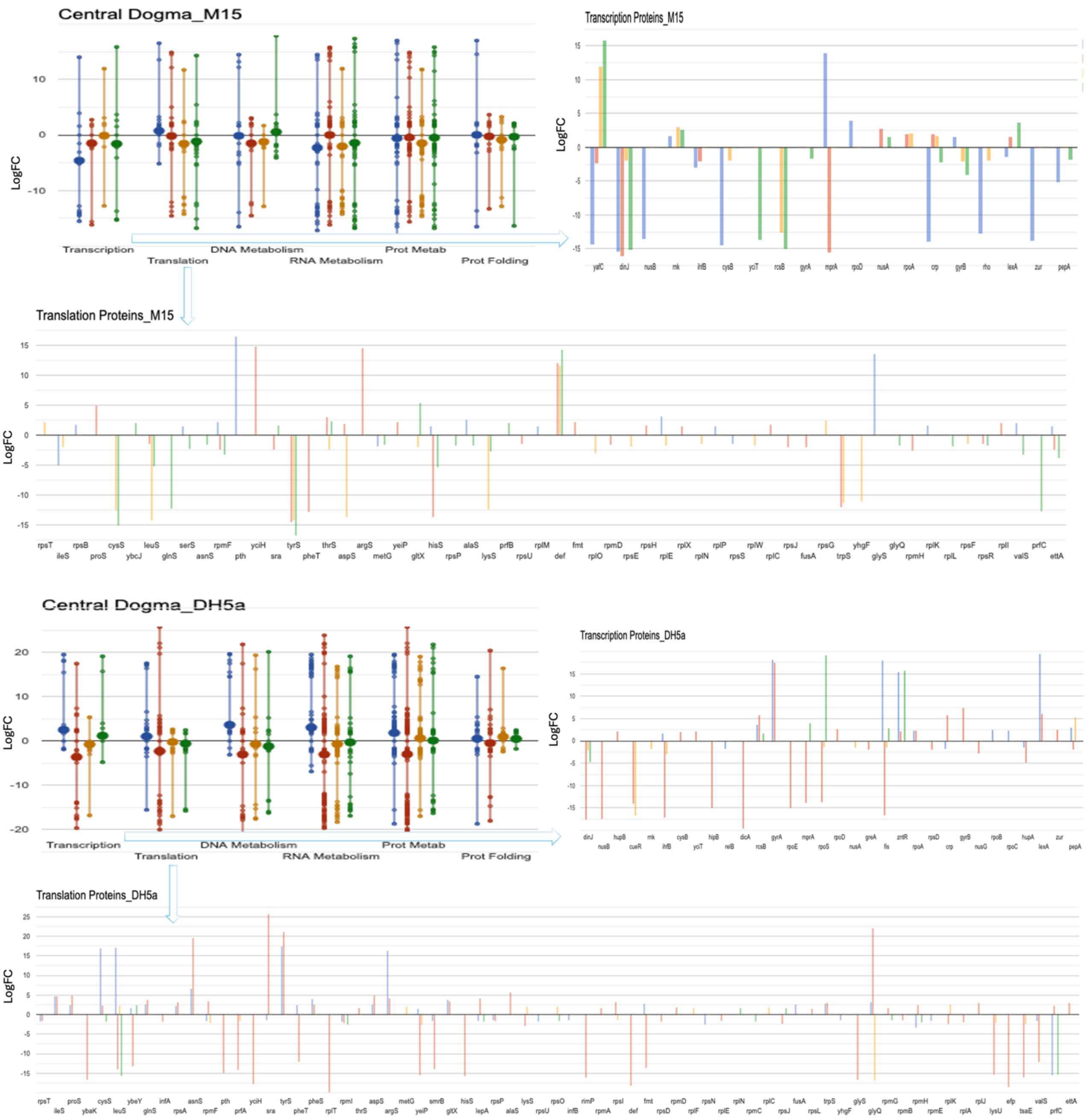

Figure S4. Overview of central dogma with differentially expressed genes and associated metabolism. Colour coding - LB: mid-log phase - Blue & late-log phase – Red; M9: mid-log phase - Yellow & late-log phase - Green.

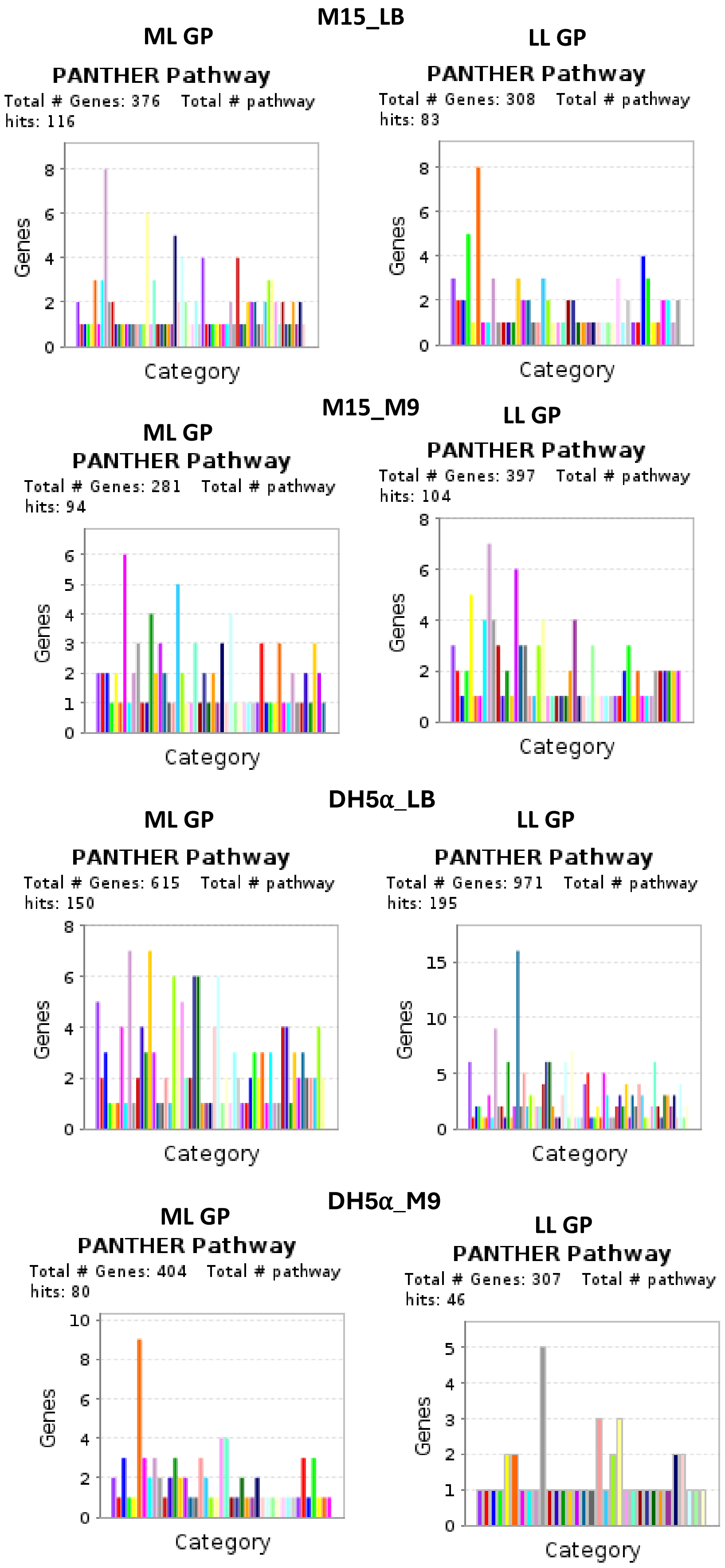

Figure S5. Gene enrichment analysis of *E. coli* M15 and DH5α in LB and M9 Medium

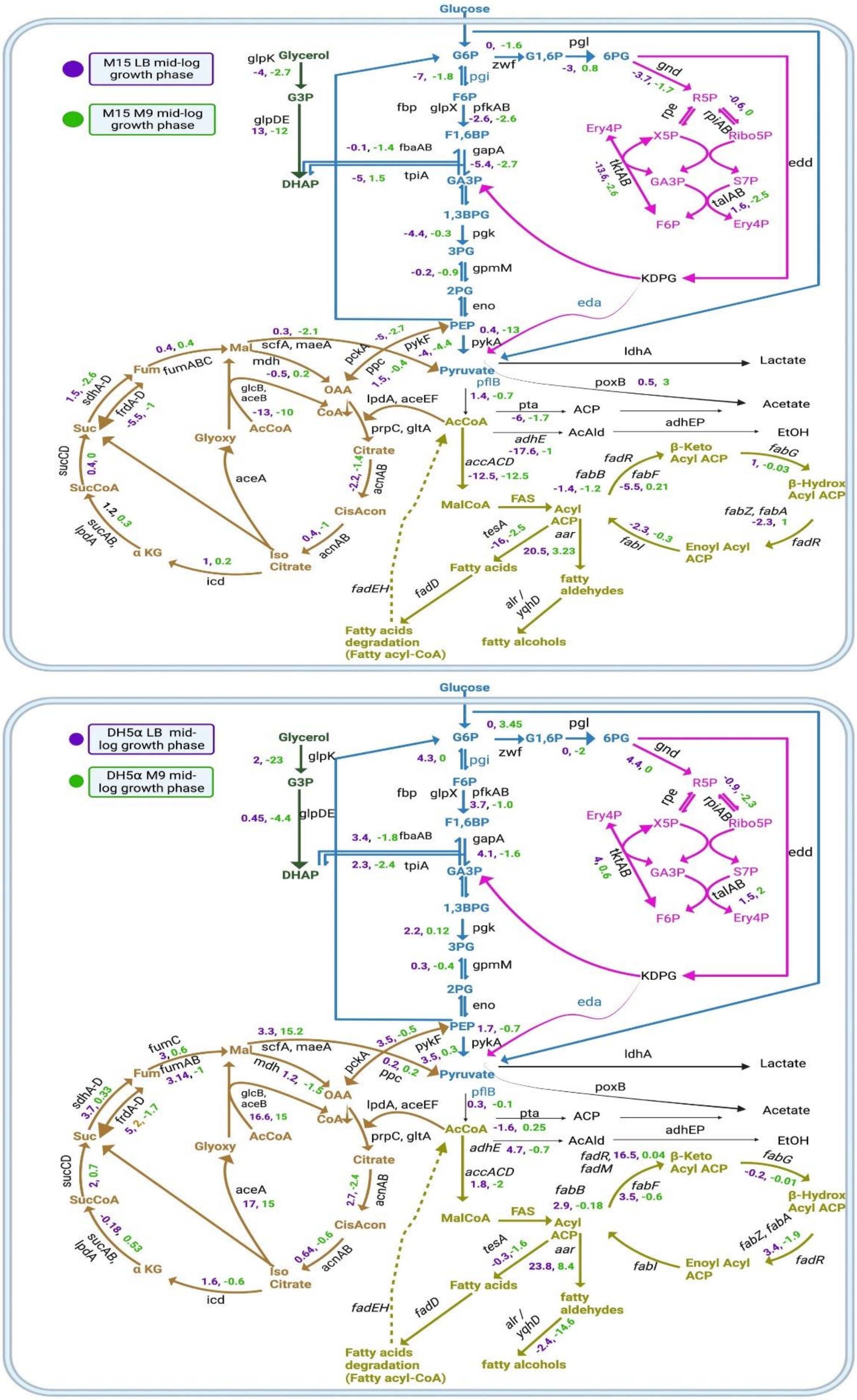

Figure S6. Insights of metabolic pathways during recombinant protein production in *E. coli* at mid-log growth phase. Numbers are representing foldchange of recombinant with respect to control.

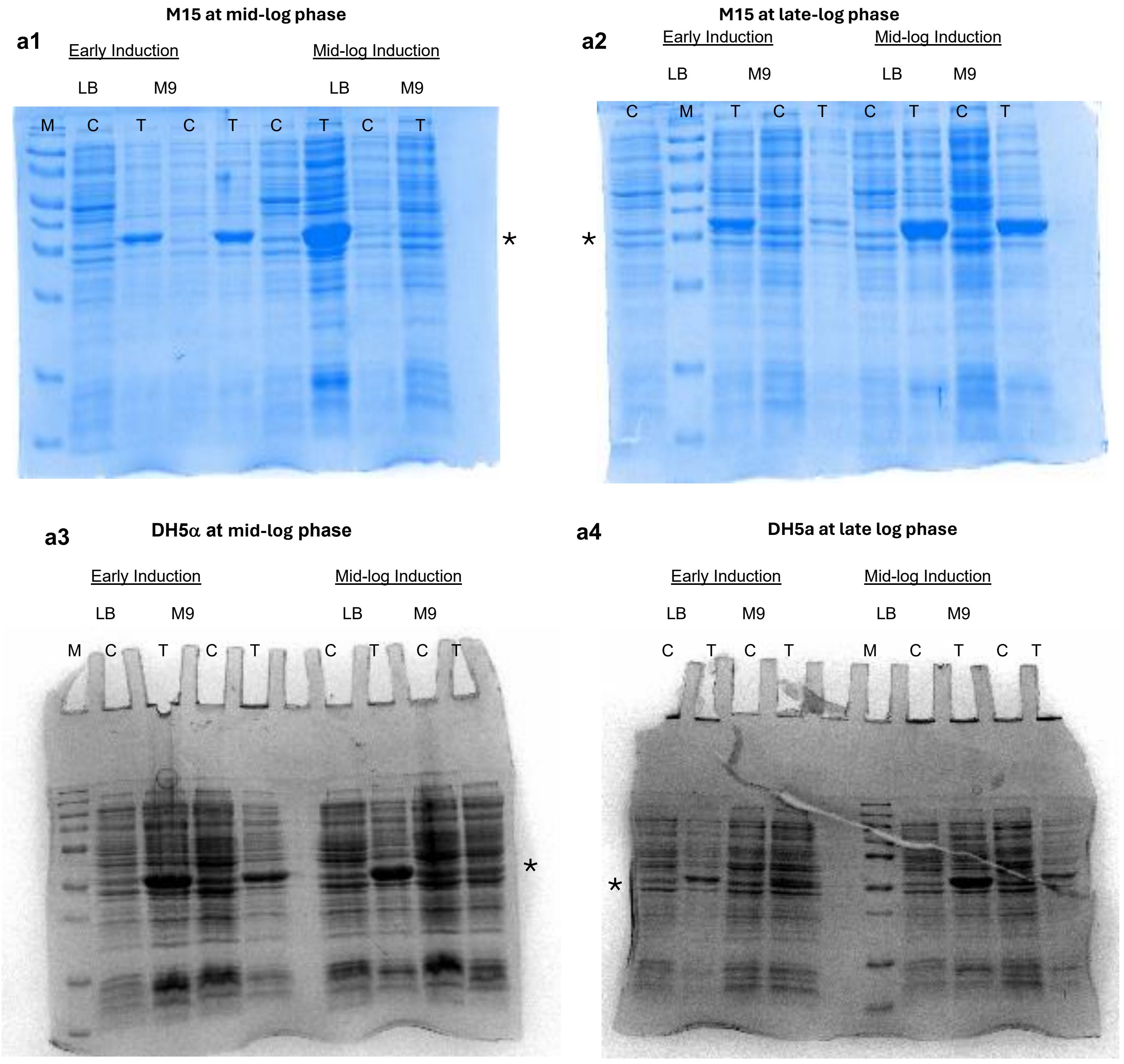

Figure S7. Expression profile of recombinant protein on SDS PAGE gel. a1- SDS PAGE for M15 at post induction mid-log phase, a2- SDS PAGE for M15 at late-log phase, a3- SDS PAGE for DH5α at post induction mid-log phase, a4- SDS PAGE for DH5α at late-log phase. LB= Luria Bertani medium; M9= Minimal modified medium; C = Control; T = Test (Recombinant strain). (*) mark indicates recombinant protein Acyl-(acyl carrier protein (ACP)) reductase (AAR).

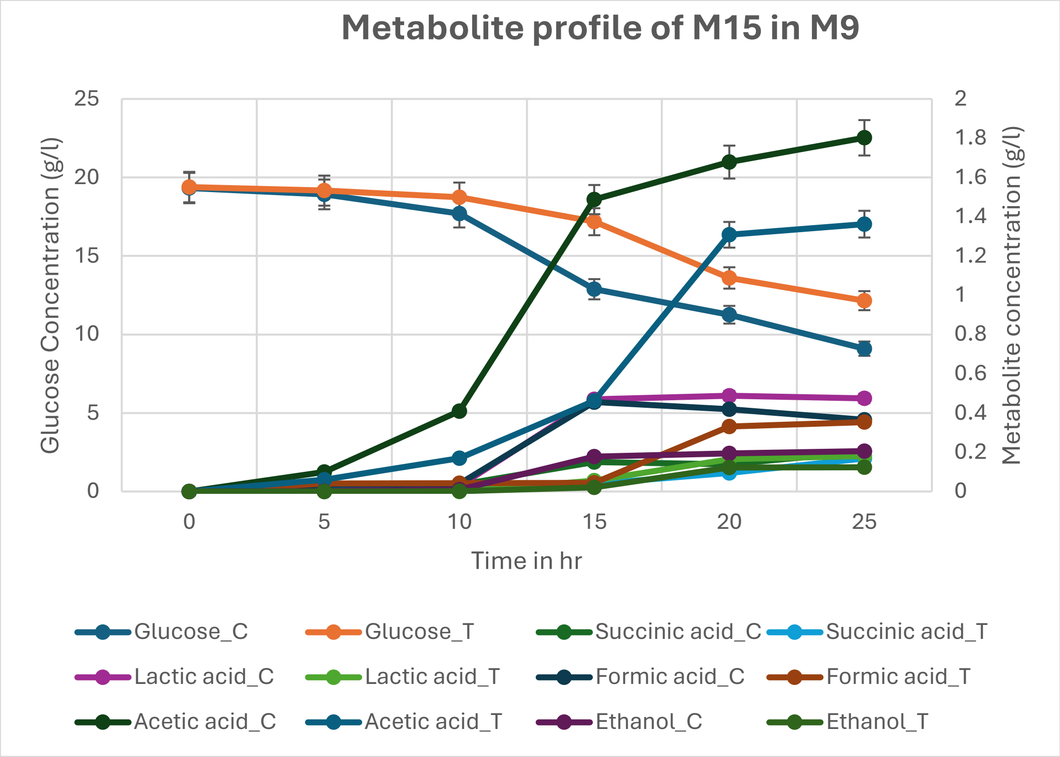

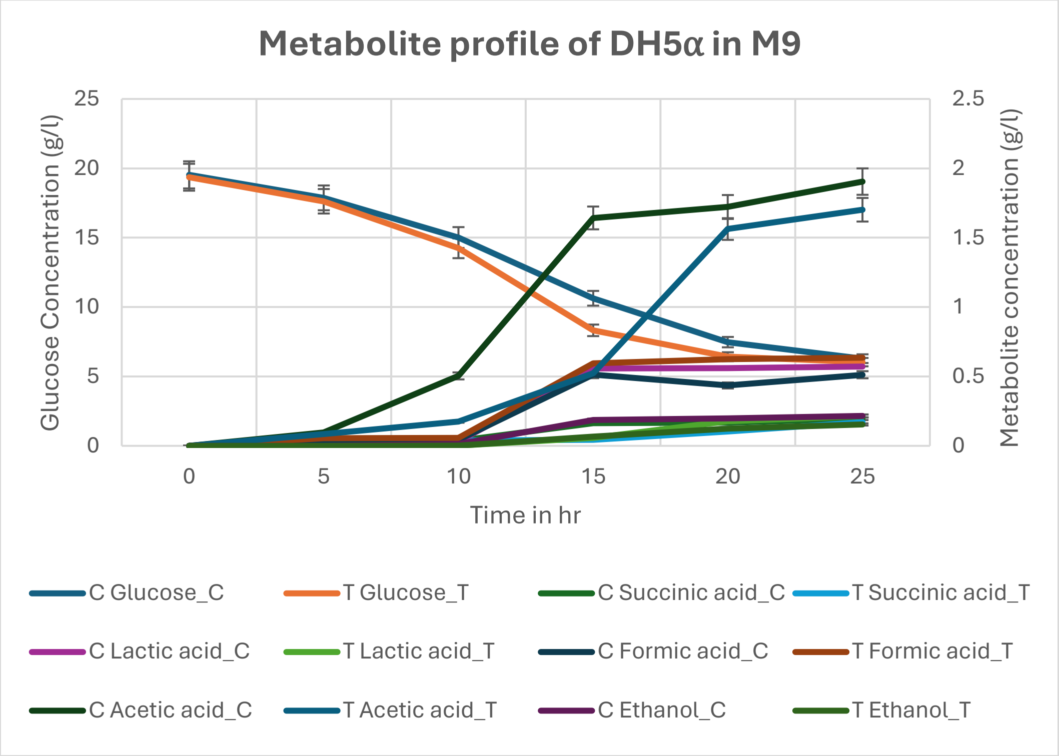

Figure S8. Substrate and product profile of *E. coli* M15 and DH5α in M9 medium
